# Antibiotic Restriction Might Facilitate the Emergence of Multi-drug Resistance

**DOI:** 10.1371/journal.pcbi.1004340

**Published:** 2015-06-25

**Authors:** Uri Obolski, Gideon Y. Stein, Lilach Hadany

**Affiliations:** 1 Department of Molecular Biology and Ecology of Plants, Tel-Aviv University, Tel Aviv, Israel; 2 Internal Medicine "B", Beilinson Hospital, Rabin Medical Center, Petah Tikva and Sackler Faculty of Medicine, Tel Aviv, Israel; University of New South Wales, AUSTRALIA

## Abstract

High antibiotic resistance frequencies have become a major public health issue. The decrease in new antibiotics' production, combined with increasing frequencies of multi-drug resistant (MDR) bacteria, cause substantial limitations in treatment options for some bacterial infections. To diminish overall resistance, and especially the occurrence of bacteria that are resistant to all antibiotics, certain drugs are deliberately scarcely used—mainly when other options are exhausted. We use a mathematical model to explore the efficiency of such antibiotic restrictions. We assume two commonly used drugs and one restricted drug. The model is examined for the mixing strategy of antibiotic prescription, in which one of the drugs is randomly assigned to each incoming patient. Data obtained from Rabin medical center, Israel, is used to estimate realistic single and double antibiotic resistance frequencies in incoming patients. We find that broad usage of the hitherto restricted drug can reduce the number of incorrectly treated patients, and reduce the spread of bacteria resistant to both common antibiotics. Such double resistant infections are often eventually treated with the restricted drug, and therefore are prone to become resistant to all three antibiotics. Thus, counterintuitively, a broader usage of a formerly restricted drug can sometimes lead to a decrease in the emergence of bacteria resistant to all drugs. We recommend re-examining restriction of specific drugs, when multiple resistance to the relevant alternative drugs already exists.

## Author Summary

Methods for minimizing antibiotic resistance are becoming more important as antibiotic resistance frequencies are rising, coupled with low discovery rates of new antibiotics. In this work we examined the practice of restricting specific drugs to be used only as 'last resort'. The goal of such restrictions is to maintain low resistance levels to certain drugs, and prevent the creation of bacteria resistant to all available treatment options. We used a mathematical model to study the impact of such restrictions, when some resistance to the unrestricted drugs is already present. We estimated the resistance frequencies of common bacteria from hospital data. We find that restricting drugs leads to increased rates of incorrect treatment, and might simultaneously lead to increased emergence of multidrug resistant bacteria. We conclude that restricting specific antibiotics should be done with caution. In some cases lifting restrictions might even delay MDR emergence.

## Introduction

Antibiotic resistance management is one of the most pressing public health issues challenging modern medicine [1]. Resistance frequency is continually rising in abundant pathogenic bacteria [2,3], entailing higher risk for patients and increased economic costs [4,5]. Especially perilous are infections of multi-drug resistant (MDR) bacteria, which can lead to higher rates of inadequate treatment and mortality [6–8], and at times are resistant to almost all available antibiotics [9,10]. Even drugs of last resort, with relatively low prescription rate, are revealed to have increasing resistance frequencies [11,12].

The increase in antibiotic resistance frequencies is a consequence of microbial evolution and adaptation, induced by vast anthropogenic antibiotic consumption and dissemination [13]. This arms-race hinges on two main forces, acting at opposite directions: resistance acquisition and spread in bacterial populations, and new antibiotic production [14]. Seeing that rates of new antibiotics' production are steadily decreasing [15] (although efforts are made to motivate the development of new antibiotics [16,17]), we are left to consider measures to impede the emergence of new resistant, and particularly MDR, bacteria. Reducing superfluous antibiotic usage, both in human consumption and veterinary usage, is currently considered the prominent course of action [18,19].

Albeit the benefit of reducing overall superfluous antibiotic usage is easy to justify and seems to have almost no drawbacks; restricting specific antimicrobial agents involves a compromise between increasing resistance to a drug saved for the most acute scenarios, versus increasing the probability of correct treatment.

The rationale of restricting specific drugs is an evolutionary one. The use of a certain antibiotic exerts selective pressure on microbes to acquire resistance to that antibiotic. However, restricting an antibiotic does not amount to complete avoidance of its usage, as it will still be used against infections resistant to all other antibiotics [20]. Thus an increase in the frequency of bacteria resistant to the common antibiotics might result in increased usage of the restricted antibiotic as well.

Recent theoretical studies support these claims: Wu et al. have shown that maintaining variation in stockpiled influenza drugs might substantially reduce resistance [21], while Joyner et al. demonstrated the benefits of developing new antibiotics and expanding the range of possible antimicrobial treatments [22].

In this work we try to assess the conditions under which a restricted antibiotic agent should be considered for broader usage. We use a mathematical model to describe the dynamics of antibiotic resistance within a hospital unit. We model three antibiotics effective against a bacterial pathogen, and bacterial strains with all combinations of resistance to these antibiotics. We concentrate on a scenario where two antibiotics are more frequently used than a third one. Therefore resistance levels to the first two antibiotics are high, while the third antibiotic has thus far been restricted to emergency use only, and the level of resistance against it is lower. This is compatible with 'last resort' antibiotics such as Vancomycin, Linezolid and Daptomycin. We compare the efficiency of antibiotic appliance strategies where one of the antibiotics is restricted, to strategies where all drugs are equally applied. We examine the impact of antibiotics restriction in the context of a commonly used antibiotic prescription strategy: mixing, wherein each drug is prescribed to each patient with equal probability [23–25]. Several works have compared mixing and other strategies [23–27]. Here, we focus on a different question: the potential effect of restricting antibiotics on multi drug resistance within the framework of mixing. We note that similar results were obtained under a 'cycling' strategy (S5 Text).

Data obtained from Rabin medical center, Israel, is used to estimate realistic single and double antibiotic resistance frequencies in incoming patients.

## Methods

### Ethics statement

The study was approved by the Helsinki Committee of Rabin Medical Center.

### The model

We have developed a mathematical model consisting of eight differential equations, describing the changes in the frequencies of patients infected with different bacterial strains within a hospital unit. The model is an extension of existing models, used for evaluating and predicting the efficiency of antibiotic treatment strategies [23,24,26,27]. Here we assume there are three antibiotics available for empiric treatment, rather than two, as in most classical models. This extension allows us to consider the contribution of both double and single resistant bacteria to the emergence of triple resistance. Frequencies of resistance can vary significantly between strategies and conditions, so incorporating different combinations of resistant types entails a more realistic depiction of the dynamics.

We denote by *S* the frequency of patients infected with bacteria susceptible to all three antibiotics; *R*_*i*_ is the frequency of patients infected with strains resistant only to antibiotic *i*, for *i* ∊ {1, 2, 3}; *R*_*i*,*j*_ is the frequency of patients infected with bacteria resistant to both antibiotics *i* and *j*, and only to these antibiotics, for (*j*, *k*) ∊ {(1, 2),(1, 3), (2, 3)}; *X* is the frequency of patients uninfected with any of the bacterial strains. We assume that strains resistant to all three antibiotics are not present yet, and only the rate of their emergence is estimated.

Each infected patient is immediately treated upon admission, with one antibiotic only, and is either cleared from infection or acquires resistance with rate *τ*. The probability of acquiring resistance to antibiotic *i* is *p*_*i*_, so that the rate of resistance acquisition upon treatment is *τp*_*i*_, and the rate of clearance is *τ*(1−*p*_*i*_).

For each strategy, *χ*_*i*_ is the fraction of patients initially receiving antibiotic *i*.

The mixing strategy consists of the application of drugs at constant probabilities throughout time. Using only two drugs, while reserving the third drug to double resistant infections alone, will be termed *mix*2. Formally:$\chi_{1}\left(mix2\right)=\chi_{2}\left(mix2\right)=\frac{1}{2},\;\chi_{3}\left(mix2\right)=0$. The application of all three drugs equally will be denoted *mix*3:$\chi_{1}\left(mix3\right)=\chi_{2}\left(mix3\right)=\;\chi_{3}\left(mix3\right)=\frac{1}{3}$.

A patient carrying an infection with a confirmed resistance to antibiotic *k* has to be treated with either antibiotic *i* or *j*. Such a patient will be treated with antibiotic *i* with probability $\chi_{i,j}^{i}$. E.g. if a patient is found (through microbiological testing) to be resistant only to antibiotic 1, under *mix*2 he will receive antibiotic 2 only $\left(\chi_{2,3}^{2}=1,\;\chi_{2,3}^{3}=0\right)$, while under *mix*3 and he will receive either antibiotic 2 or 3 with equal probability $\left(\chi_{2,3}^{2}=\;\chi_{2,3}^{3}=\frac{1}{2}\right)$. For simplicity, we do not differentiate between patients acquiring resistance during treatment and those admitted with resistance.

An infection resistant to antibiotic *i* is assumed to be unaffected by treatment with it. Patients treated incorrectly—i.e., with an antibiotic that their infection is resistant to—are switched to a correct treatment after a delay period [27]. This is modeled by assigning a slower clearance rate to a proportion of the patients carrying resistant infections. The rate of recovery when the delay applies is given by *τd*, so that for a delay of *D* days, we define $d=\frac{1}{1+\tau D}$. Thus, for example, a fraction *χ*_*1*_ of patients of class *R*_1_ will have a recovery rate of *τd* while a fraction *χ*_*1*_ + *χ*_*3*_ of that class will have a recovery rate *τ*. A fraction $\chi_{1}\chi_{2,3}^{2}$ will be treated with antibiotic 2, after a delay, and their rate of resistance acquisition will be d*τp*_2_. *γ* is the rate of spontaneous recovery due to the response of the patient's immune system; *β* is the rate of bacterial transmissions resulting in infection (for simplicity, super-infection is neglected). Since we assume the bacterial infection considered is not the main reason of hospitalization, patients leave the hospital, or die, at a rate proportional to their frequency, with the proportion constant *c*. This common assumption allows us to model constant hospital occupancy.

*λ*_*V*_ is the rate of patients of class *V* entering the hospital (e.g., *λ*_*S*_ is the rate of patients admitted to the hospital with bacteria susceptible to all three antibiotics). We assume a constant population size, so that the total admission rate is equal to the discharge rate, namely:$\lambda_{X}+\lambda_{S}+\lambda_{R_{1}}+\lambda_{R_{2}}+\lambda_{R_{3}}+\lambda_{R_{1,2}}+\lambda_{R_{1,3}}+\lambda_{R_{2,3}}=c$. For convenience, we also define the auxiliary parameters $f_{V}=\frac{\lambda_{V}}{c-\lambda_{x}},\;\text{for}\;\;V\in \left\{R_{1},R_{2},R_{3},R_{1,2},R_{1,3},R_{2,3},S\right\}$. *f*_*V*_ is the frequency of incoming patients carrying a resistant pathogen of class *V* out of all patients carrying the pathogen, and will be used throughout this work. The model parameters, interpretations, values used and references (taken from [20,28–35]) are given at Table 1.

**Table 1:** Parameters, their meaning and values.

Parameter	Meaning	Value
*β*	Rate of bacterial transmission	0.01–0.5 day^-1^ [28,29]
*p_i_*	Probability of developing resistance to antibiotic *i* during treatment	0–0.3 [30,31]
*γ*	Rate of clearance due to the patient's immune system	0.03 day^-1^
*τ*(1−*p_i_*)	Rate of clearance due to antibiotic *i*	$\frac{1}{7}$ day^-1^ [20]
*c*	Rate of patient turnover (length of stay^-1^)	0.1 day^-1^ [28]
*λ_V_*	Entrance rates of patients of class V	Varies (day^-1^ units)
*f_V_*	Resistance frequency of incoming patients, of the class V	Varies
$f_{R_{3}}$	Resistance frequency of incoming patients, of the restricted antibiotic (antibiotic 3)	0.001 [32,33]
*d*	Delay of correct treatment factor	0.778 [34]
*π*	Hospital size relative to community	$\frac{1}{300}$ [35]
*Χ_i_*	Fraction of patients initially treated with antibiotic *i*	Determined by strategy
$\chi_{i,j}^{i}$	Fraction of patients treated with antibiotic *i*, for which resistance to antibiotic *k* ≠ *i*, *j* was established.	Varies
*t*1–*t*0	Duration of time the dynamics are tested for.	20–100 years

The full model is comprised of the following differential equations (for *i* ∊ {1, 2, 3},(*j*, *k*) ∊ {(1, 2),(1, 3), (2, 3)}, *i* ≠ *j* ≠ *k*):

$$\begin{array}{l} X=1-S-R_{1}-R_{2}-R_{3}-R_{1,2}-R_{1,3}-R_{2,3}\;\\ \dot{S}=\lambda_{S}-cS+\beta SX-\left(\tau +\gamma \right)S\;\\ {\dot{R}}_{i}=\lambda_{R_{i}}-R_{i}\left(c+\gamma \right)+\beta R_{i}X-\tau \left(\chi_{j}+\chi_{k}+\chi_{i}d\right)R_{i}+\tau \chi_{i}Sp_{i}\;\\ {\dot{R}}_{j,k}=\lambda_{R_{j,k}}-R_{j,k}\left(c+\gamma \right)+\beta R_{j,k}X-\tau \left(\chi_{i}+\left(\chi_{j}+\chi_{k}\right)d\right)R_{j,k}+\\ \left(\chi_{k}+\chi_{j}d\chi_{i,k}^{k}\right)\tau R_{j}p_{k}+(\chi_{j}+\chi_{k}d\chi_{i,j}^{j})\tau R_{k}p_{j} \end{array}$$ (1)

**Fig 1:**
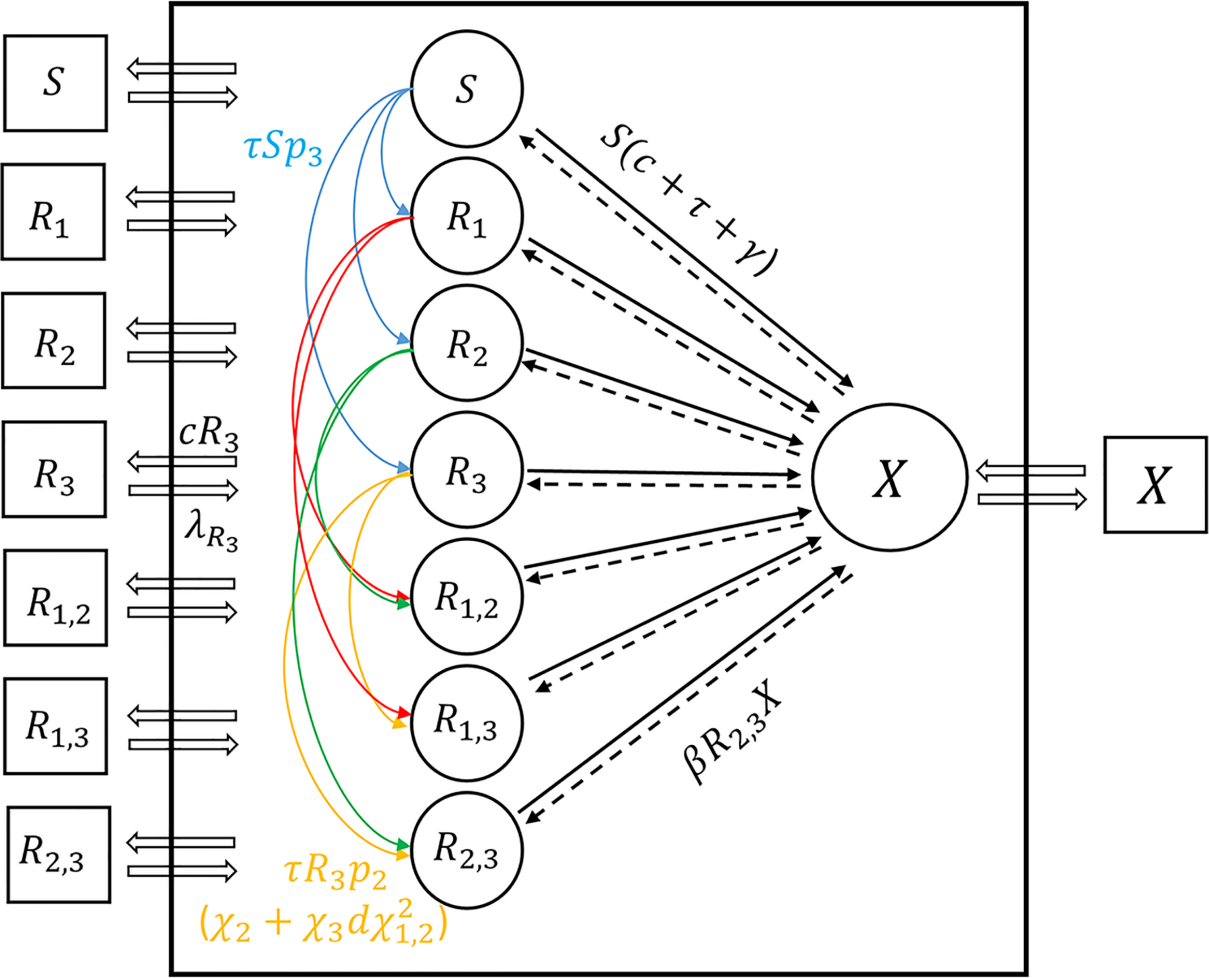
An illustration of the dynamic system presented in Eq 1. The black frame represents the hospital, with hollowed arrows signifying patients moving in and out between the hospital and the community; circles representing patient frequencies within the hospital, and squares representing the frequencies of patients infected with bacterial strains in the community, with respect to variable names within the shapes (see methods). Colored arrows show the direction of resistance acquisition due to treatment; solid black arrows are recovery from infected to cleared states, while dashed lines are infections. Several arrows are marked with the corresponding rates of flow between variables.

An illustration of the dynamics is given in Fig 1.

We derived an analytical approximation of the equilibrium state of (Eq 1) (see S1 Text for the analysis and agreement between the approximation and the numerical solution of the equilibrium).

We note that our model can be reduced to a two-drug model, if all the parameters and variables pertaining to double resistance and to resistance to the third drug are set to zero. We can model a strategy where only the first drug is used, except for treatment of resistant infections, and two alternative strategies of mixing and cycling both drugs (see supplementary material III).

### Adding community feedback

We also include in our model the impact of different strategies on resistance rates in the community. Given that the mean patient length of stay is $\frac{1}{c}$ days, then on average a fraction *c* of the patients leave (and enter) each day. We will denote the ratio of the hospital size to the size of the community it serves as *π*. The number of hospital beds per capita can serve as an estimate for *π*, and the estimate is usually within the range $\frac{1}{1000}\leq \pi \leq \frac{1}{200}$, with $\pi \approx \frac{1}{300}$ for developed countries [35].

We assume that the total incidence of infection by the modeled pathogen is constant and model only the change in the distribution of different types of resistant infections within the community.

This is modeled by changing the frequencies of different incoming patients (which are proportional to the entrance rates) from constant in (Eq 1) to dynamic. We add to (Eq 1) the following ODEs:

$${\dot{f}}_{V}=\left(\frac{V}{1-X}-f_{V}\right)c\pi$$

For *V* ∊ {*S*, *R*_1_, *R*_2_, *R*_3_, *R*_1,2_, *R*_1,3_, *R*_2,3_}.

Thus the entrance rates of all infected patients ($\lambda_{S},\lambda_{R_{1}},\lambda_{R_{2}},\lambda_{R_{3}},\lambda_{R_{1,2}},\lambda_{R_{1,3}},\lambda_{R_{2,3}}$) become dynamic and are affected by the efflux of the various strains to the community. The chosen method of modeling assumes that the discharged patients are replacing patients in the community (which are admitted into the hospital) at rate *c*, with the weight of each discharged patient, relative to the community, being *π*. Although models explicitly computing the dynamics in the community exist [36], we chose a simplified model that captures the main component, of varying strength of influence of the hospital on the community (*π*).

### Measuring the strategies' efficiency

We employ two criteria to measure the efficiency of each treatment strategy:

First, we measure the mean frequency of incorrectly treated patients. This measure was chosen since inadequate treatment leads to higher mortality, in single-resistant and MDR bacteria [6,37,38]. The mean frequency of initially incorrectly treated patients under a strategy *σ* ∊ {*mix*2, *mix*3}, for *i* ∊ {1, 2, 3},(*j*, *k*) ∊ {(1, 2),(1, 3), (2, 3)}, *i* ≠ *j* ≠ *k*), is:

$$Incorrect\left(\sigma \right)=\frac{1}{t_{1}-t_{0}}\int_{t_{0}}^{t_{1}}\left(\sum_{i}R_{i}\left(t,\sigma \right)\chi_{i}\left(\sigma \right)+\sum_{j,k}\left(R_{j,k}\left(t,\sigma \right)\left(\chi_{j}\left(\sigma \right)+\chi_{k}\left(\sigma \right)\right)\right)\right)dt$$

Where *t*_0_, *t*_1_ are the times of beginning and end of measuring, respectively. Second, we measure the emergence rate of triple resistant bacteria. We estimate triple resistance emergence as proportional to the number of double resistant infections treated with the only antibiotic they are susceptible to. Therefore, the mean emergence rate of triple resistance under a treatment strategy *σ* is proportional (with the proportion constant being the clearance rate) to:

$$Triple\left(\sigma \right)\propto \frac{1}{t_{1}-t_{0}}\int_{t_{0}}^{t_{1}}\left(\sum_{j,k}R_{j,k}\left(t,\sigma \right)\left(\chi_{i}\left(\sigma \right)+d\left(\chi_{j}\left(\sigma \right)+\chi_{k}\left(\sigma \right)\right)\right)p_{i}\right)dt$$

We used several methods to decide which parameters are most influential in our system. First, we used the analytical approximation of the equilibrium state of the ODE system without the community feedback to see the influence of various parameters on the system (S1 Text). In addition, we performed a parameter sensitivity analysis (PSA) of the system (S4 Text). The result of the PSA suggested that the resistance frequencies of incoming patients are the most influential parameters on the dynamics. We thus focus on these parameters and use estimates from clinical data for their values. We also performed pairwise comparisons of parameters to determine the effect of their interaction on the emergence of triple resistance (S2 Text).

To further investigate the implications of our model, we estimated realistic resistance frequencies of incoming patients, using data gathered from patients in Rabin medical center, Israel, dating 2009–2012, that had positive blood cultures for bacteremia. We used antibiotic resistance profiles of blood cultures obtained up to 48 hours after patient admission, to avoid estimating resistant infections acquired within the hospital. Antibiotics co-occurring in resistance tests were divided to all possible pairs, and frequencies of single and double resistance were estimated (corresponding to *f*_1_, *f*_2_ and *f*_1,2_). Only observations with ≥50 instances of testing for resistance of both antibiotics were considered, resulting in 73 pairs of antibiotics (see S1 Table).

## Results

We concentrate on a scenario in which two antibiotics (in our model, antibiotics 1 and 2) are frequently used, and resistance levels to them are not low. In contrast, antibiotics 3 is assumed to have thus far been restricted for emergency use only, and the frequency of resistance to it is still low. We examine the change prompted by enabling the broad usage of antibiotic 3 under the mixing strategy.

The mixing strategy can be thought of as the default strategy applied in hospitals, when physicians are prescribing antibiotics from a predetermined set, with no specific time-dependent preference. We use our mathematical model to examine the effect of mixing the three antibiotics available versus mixing only two and restricting the third (*mix*3 and *mix*2, respectively).

**Fig 2:**
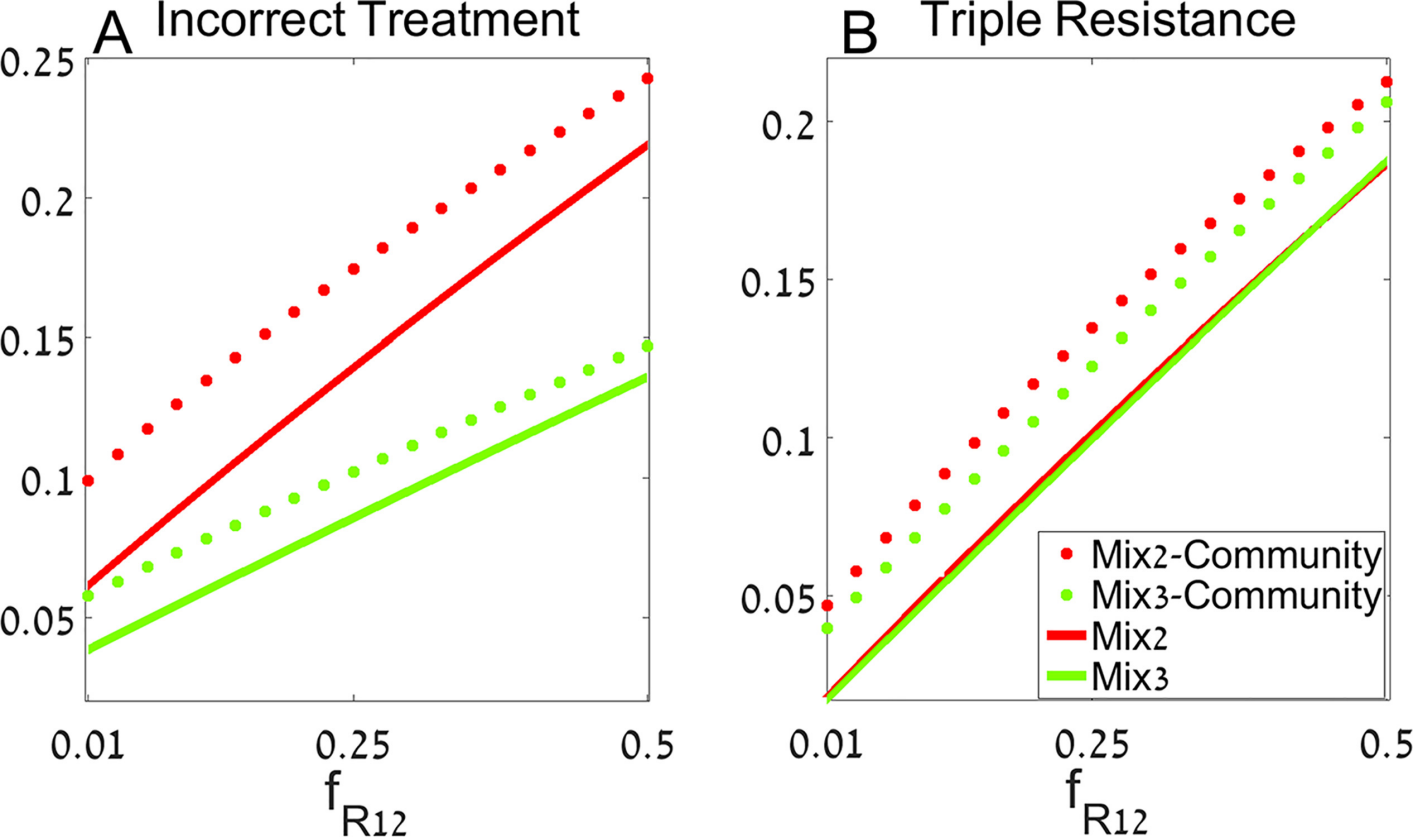
The mixing strategy for varying double resistance frequencies. We measure the fraction of incorrect treatment (A), and the rate of triple resistance emergence (B) for varying levels of double resistance to the commonly used antibiotics ($f_{R_{1,2}}$). Red curves represent the results under mixing 2 antibiotics and restricting antibiotic 3 (*mix*2), while green curves are the results under mixing 3 antibiotics (*mix*3). Dotted and solid lines are the results of the model with and without community feedback, respectively. Parameters are $f_{R_{1}}=\;f_{R_{2}}=0.1,\beta =0.3,\;\lambda_{X}=0.07,p_{1}=p_{2}=p_{3}=0.07$ $\;\lambda_{S}=c-\lambda_{X}-\lambda_{R_{1}}-\lambda_{R_{2}}-\lambda_{R_{3}}-\lambda_{R_{1,2}}-\lambda_{R_{1,3}}-\lambda_{R_{2,3}}$, and the rest are given at Table 1. The system is simulated for 20 years and other parameter values are given in the text.

We start by examining the frequency of double and single resistance of incoming patients' infections, with respect to the commonly used antibiotics ($f_{R_{1,2}}$ and $f_{R_{1}},f_{R_{2}}$, respectively). Whereas resistances to different antibiotics might be independent ($f_{R_{1,2}}\approx f_{R_{1}}f_{R_{2}}$), selection for resistance could drive the double resistance frequencies to higher values than expected by random. In Fig 2 we set the values of single resistance frequency to $f_{R_{1}}=\;f_{R_{2}}=0.1$, while varying $f_{R_{1}}f_{R_{2}}\leq f_{R_{1,2}}\leq 5f_{R_{1}}$ on the horizontal axis.

The vertical axis presents the mean fraction of incorrectly treated patients (Fig 2A), and mean triple resistance emergence rate (Fig 2B) over 20 years. We plot the results of the model assuming no effect of hospital resistance frequencies on the community (solid lines) and of the model with community feedback (dotted lines). We set the double resistance frequencies for the third restricted antibiotic to $f_{R_{3}}=f_{R_{2,3}}=f_{R_{1,3}}$, as a conservative estimation. Similar results are obtained when resistance to antibiotic 3 is assumed to be independent of resistance to antibiotics 1 and 2. Other parameters are $\beta =0.3,\;\lambda_{X}=0.07,p_{1}=p_{2}=p_{3}=0.07,\;\lambda_{S}=c-\lambda_{X}-\lambda_{R_{1}}-\lambda_{R_{2}}-\lambda_{R_{3}}-\lambda_{R_{1,2}}-\lambda_{R_{1,3}}-\lambda_{R_{2,3}}$. References for the parameter values used are given in Table 1.

We can see that introducing the previously restricted drug to common usage decreases the prevalence of incorrect treatment in our model, whether regarding community feedback or not (Fig 2A). This is intuitive, as a relatively small fraction of the incoming patients is resistant to antibiotic 3, and thus using it leads to a decreased probability of incorrect treatment.

When considering the emergence of triple resistance, the picture is more complex. On the one hand, the unrestricted usage of the antibiotic 3 limits the spread of double resistance, most common of which is resistance to antibiotics 1 and 2. On the other hand, more frequent usage of antibiotic 3 causes more patients to acquire resistance to it during treatment. This can be seen when examining the triple resistance emergence (*Triple*(*σ*)). If we assume that resistance to both common drugs is significantly higher than resistance to the third drug, then *Triple*(*σ*) is dominated by the term:

$$E\left(\sigma \right)=R_{1,2}\left(t,\sigma \right)\left(\chi_{3}\left(t,\sigma \right)+d\left(\chi_{1}\left(t,\sigma \right)+\chi_{2}\left(t,\sigma \right)\right)\right)p_{3}$$

The homogeneity in time of the mixing strategies allows for a simple analysis. *E*(*σ*) under *mix*2 becomes

$$E\left(mix2\right)=R_{1,2}\left(t,mix2\right)\left(0+d\left(\frac{1}{2}+\frac{1}{2}\right)\right)p_{3}=dR_{1,2}\left(t,mix2\right)p_{3}$$

while for mix 3 it becomes

$$E\left(mix3\right)=R_{1,2}\left(t,mix3\right)\left(\frac{1}{3}+d\left(\frac{1}{3}+\frac{1}{3}\right)\right)p_{3}=\frac{2d+1}{3}R_{1,2}\left(t,mix3\right)p_{3}.$$

Thus the condition for reducing triple resistance emergence under *mix*3 is approximately *E*(*mix*2) > *E*(*mix*3), which implies that triple resistance is lower under *mix*3 at a time point *t* if

$$\frac{R_{1,2}\left(t,mix2\right)}{R_{1,2}\left(t,mix3\right)}>\frac{2}{3}+\frac{1}{3d}.$$ (2)

Thus *mix*3 improves as switching to a correct treatment is done faster. Had patients been immediately treated with an effective drug (*d* = 1), the strategy best minimizing *R*_1,2_ would have reduced triple resistance emergence. When a delay occurs, *mix*3 will have to reduce *R*_1,2_ by a factor of $\frac{2}{3}+\frac{1}{3d}$, relative to *mix*2. This is a lower bound (i.e. *mix*3 will have to reduce double resistance more than the mentioned factor) since the neglected terms (*R*_1,3_, *R*_2,3_) are higher under *mix*3 than under *mix*2. Furthermore, under mix3 *R*_1,3_, *R*_2,3_ might increase with time due to selective pressure, leading to a substantial underestimation of the bound in (Eq 2).

We should note that if one strategy is able to decrease both incorrect treatment and triple resistance emergence more efficiently than another, then this strategy should be preferred. However, when one strategy is only better at reducing incorrect treatment, while producing higher triple resistance, the costs and benefits of the two effects should be considered.

Furthermore, we examined the effects of various parameters on the emergence of triple resistance under *mix*3 relative to *mix*2 (S4 Text). We note that triple resistance emergence can be decomposed to two main components: the resistance emanating from the common double resistant strain (*R*_1,2_), mainly affected by infection; and from the rare double resistant infections (*R*_1,3_ + *R*_2,3_), which are usually a result of resistance acquisition. Typically, *mix*3 decreases *R*_1,2_ and increases *R*_1,3_ + *R*_2,3_, in comparison to *mix*2.

The parameters can act in opposing ways on these two terms.

High transmission rates (*β*) increase the effectiveness of *mix*3, since the main advantage of using the restricted drug is inhibiting the spread of *R*_1,2_ (Fig A in S4 Text). The opposite is true for the patient turnover rate (*c*): high turnover limits the potential time for transmission, and requires higher transmission rates for *R*_1,2_ to be substantial and for *mix*3 to be effective (Fig C in S4 Text). High antibiotic clearance rates (*τ*) increase the advantage of *mix*3, under which *R*_1,2_ patients have a higher probability of being correctly treated (Fig A and B in S4 Text. Similar results are obtained when the rate of clearance *τ* varies while the rate of acquisition *τp*_*i*_ is kept constant: Fig E in S4 Text). These parameters act similarly on *R*_1,3_ + *R*_2,3_, but the relative weight of *R*_1,2_ is usually higher than that of *R*_1,3_ + *R*_2,3_. Thus high transmission rates, low turnover rates, and high clearance rates are conditions where restriction might be inefficient for reducing triple resistance emergence. However, antibiotic clearance rates also have a strong interaction with the delay of correct treatment (1/*d*). When the delay of treatment is short but realistic—about two days ($d\approx \frac{1}{1+2\tau }$)—*mix*3 is more efficient than *mix*2 even for low clearance rates (Fig B in S4 Text). This corresponds to the result obtained above in (Eq 2).

The probabilities of resistance acquisition (*p*_1_, *p*_2_, *p*_3_) also play a role in the dynamics' outcome. As expected, *mix*3 will be less effective if resistance to antibiotic 3 is easily acquired—high *p*_3_ relative to *p*_1_, *p*_2_ (Fig D in S4 Text).

**Fig 3:**
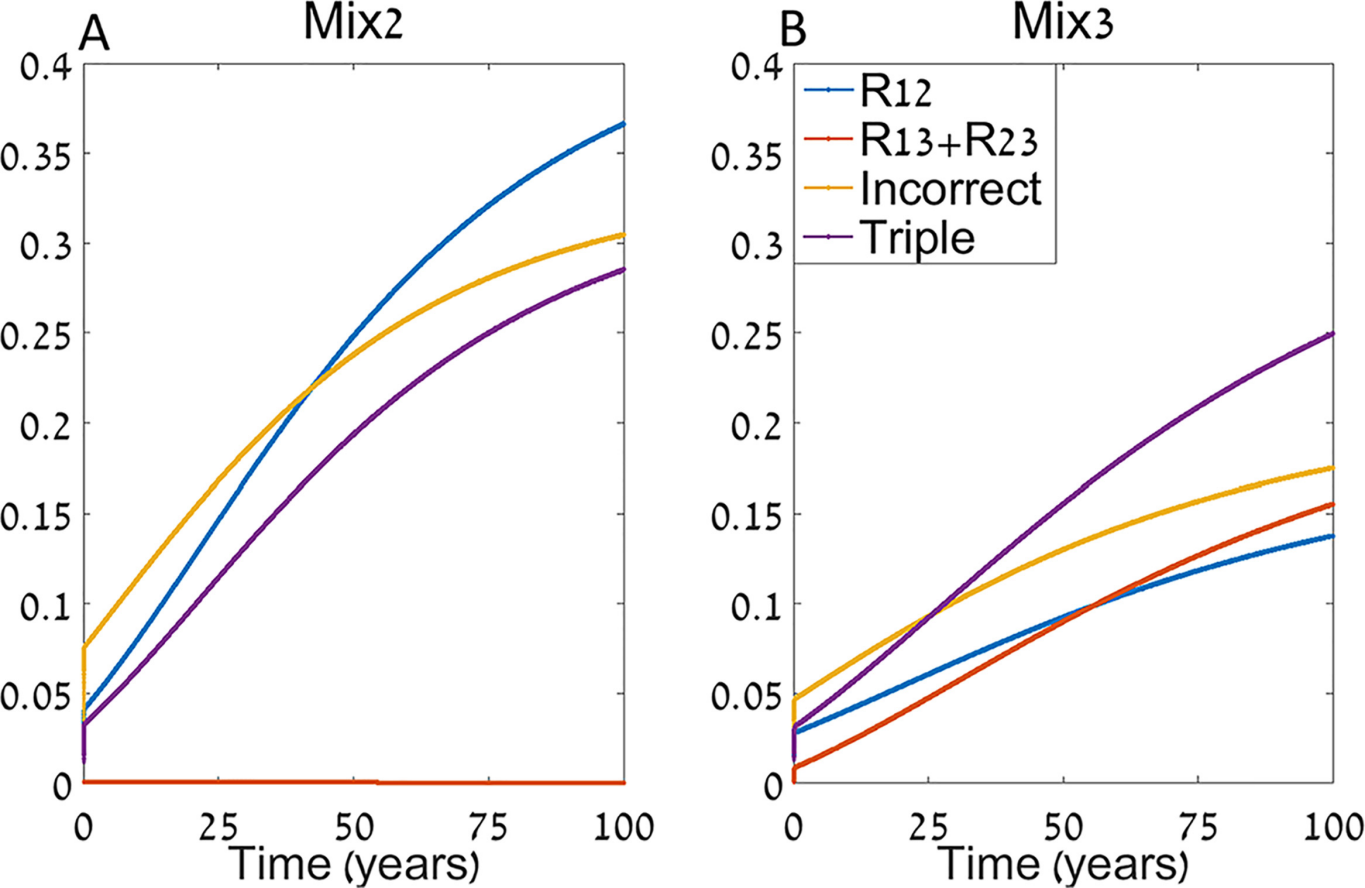
Time series of *mix*2 (A) and *mix*3 (B). We plot the frequencies of double resistant infections resistant to the third antibiotic (*R*_1,3_ + *R*_2,3_), the double resistant infections resistant to the two commonly used antibiotics (*R*_1,2_), the measured incorrectly treated patients, and the emergence of triple resistance. The model is simulated for an extended period of time (100 years) to capture long term effects and the rest of the parameters are as in Fig 2.

Another important factor that can affect the hospital dynamics is the feedback from the community. In Fig 3 we again decompose the frequency of resistant strains to *R*_1,2_ and *R*_1,3_ + *R*_2,3_, and compare the levels of incorrect treatment and triple resistance emergence for *mix*2 (Fig 3A) and *mix*3 (Fig 3B).

The parameters used are as in Fig 2, with *f*_1,2_ = 0.5 *f*_1_. One might presume that *mix*3 will be inefficient in the long term, as it results in increased *R*_1,3_ + *R*_2,3_ over time. In contrast, the dynamics show that the rapid increase of *R*_1,2_ under *mix*2 might be a more serious pitfall under these parameters, and the sum of all double resistant infections is lower under *mix*3 than under *mix*2. As a result *mix*3 stays more efficient than *mix*2 for extended periods of time. Decreasing the number of hospital beds per capita relative to the community (*π*) reduces this effect, with *π* = 0 equivalent to the model without community.

**Fig 4:**
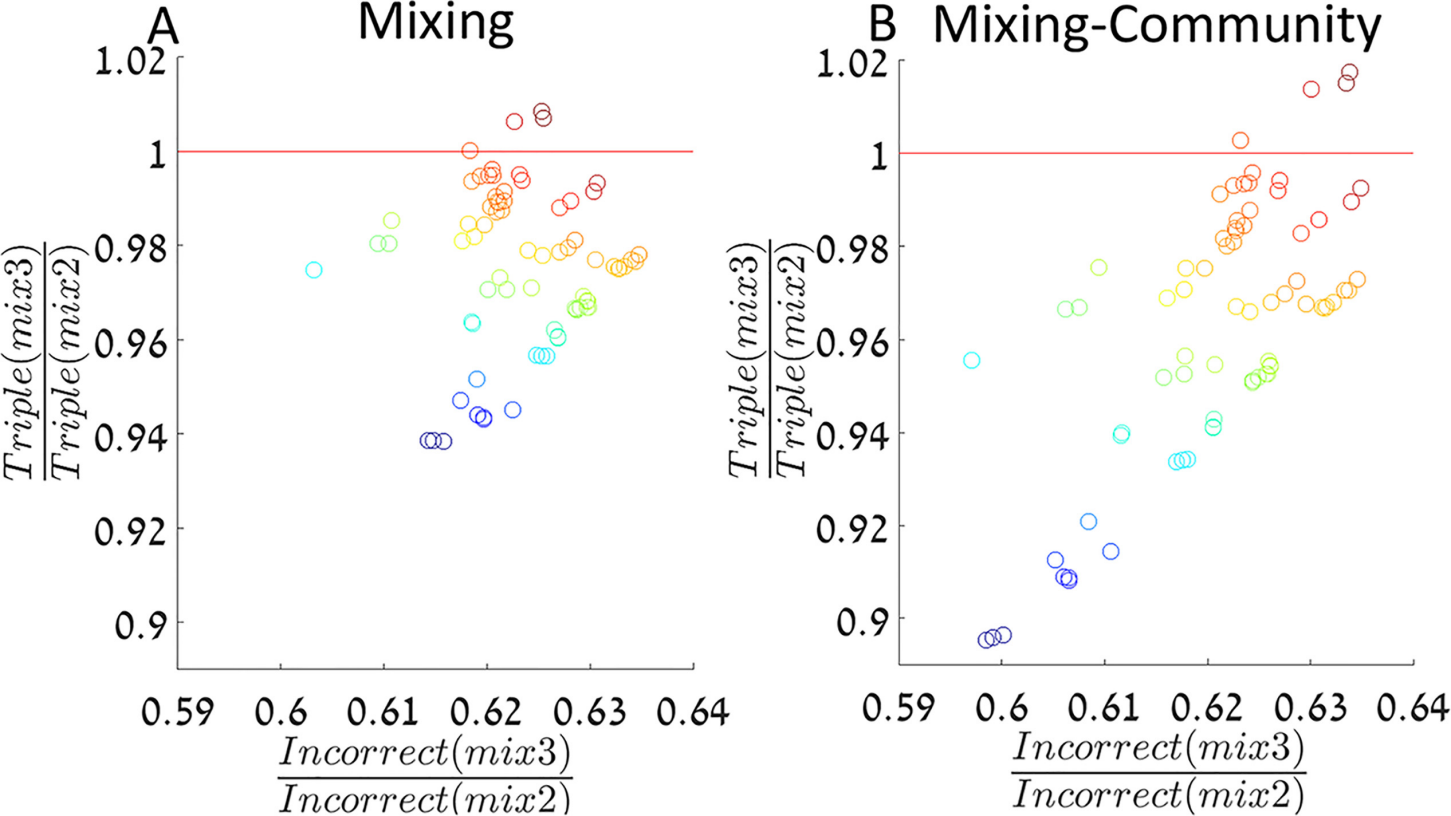
The mixing strategy for estimated resistance frequencies. For each data point, its location on the X axis represents the predicted ratio of incorrect treatment under *mix*3 relative to *mix*2 according to our model, and its location on the Y axis represents the predicted ratio of triple resistance emergence. A red line is drawn where the strategies inhibit triple resistance equally well, so below the line *mix*3 reduces both incorrect treatment and triple resistance emergence more efficiently than *mix*2. Panels A and B present the results of the models without community feedback and with it, respectively. Antibiotic resistance frequencies among incoming patients are estimated from data (see Methods and S1 Table). The color indicates the estimated resistance frequencies to the common antibiotics ($f_{R_{1}}+f_{R_{2}}+f_{R_{1,2}}$). The system is simulated for 20 years and the rest of the parameters are as in Fig 2.

To further investigate the implications of our model, we estimated single and double resistance frequencies of the common drugs (*f*_1_, *f*_2_ and *f*_1,2_) from patients in Rabin medical center, Israel, dating 2009–2012 (see Methods). We simulated our model using the estimated resistance frequencies among incoming patients, with the rest of the parameters as in Fig 2. Fig 4 presents the ratios of incorrectly treated patients and triple resistance emergence, in *mix*3 relative to *mix*2, for both the model without community feedback (Fig 4A) and with community feedback (Fig 4B).

The colors represent the sum of the estimated resistances in each sample ($f_{R_{1}}+f_{R_{2}}+f_{R_{1,2}}$). We can see that *mix*3 reduces incorrect treatment by ~40% relative to *mix*2 (Fig 4, horizontal axis), and has contrasting effects on triple resistance emergence: Albeit *mix*3 increases triple resistance emergence rate by about 1% for some samples, it usually reduces it by up to 10%, relative to *mix*2 (Fig 4, vertical axis). We see that under low levels of resistance among the infections of incoming patients, *mix*3 is strictly preferred over *mix*2—it reduces both triple resistance emergence and incorrect treatment. When higher levels of resistance are present, the dynamics may change so that refraining from treating with antibiotic 3, except when the other two antibiotics fail, decreases triple emergence. This is once again the trade-off between effective short term treatment and the risk of creating new MDR bacteria. We can also see that both ratios of incorrect treatment and triple resistance emergence rates tend to be smaller, indicating a stronger preference for *mix*3, when the hospital population has an effect on the community (Fig 4, compare panel A to panel B). This occurs due to the quick rise of *R*_1,2_ under *mix*2, and its influence on the community resistance, which is moderated under the more diverse treatment of *mix*3.

## Discussion

In this work we examined whether restricting the usage of certain antibiotic agents, i.e. keeping them as 'last resorts', is effective in inhibiting the spread of MDR bacteria. We developed a model describing hospital dynamics of a bacterial pathogen potentially treated by three available antibiotics. We assumed that the pathogen is treated by two commonly used drugs, and a third drug is only used when an infection is resistant to both common drugs. We examined the scenario where the common drugs have been used for a long enough period, so that resistance to each of the drugs, and double resistance to both of them, is not very uncommon. We tested the efficiency of restricting versus using the third drug extensively, for a common antibiotic usage strategy: mixing. The criteria for efficiency were defined as the mean number of incorrectly treated patients, and the rate of triple resistance emergence. We considered dynamic resistance frequencies in the community, affected by patients discharged from the hospital, as well as constant frequencies.

We conclude that introducing the previously restricted antibiotic to broad usage always decreases incorrect treatment, and can even simultaneously reduce the emergence of triple resistance sometimes.

A similar question can be asked considering only two antibiotics; one commonly used and one restricted. In such a simple model, the usage of two antibiotics will decrease the level of incorrect treatment, and under some conditions also the resistance emergence (see S3 Text). However, the simple model does not apply when double resistant strains are already present, as is the case for many antibiotics (see S1 Table). One major difference between the models is the rate of conversion from the sensitive type to the multiply resistant type. More steps of resistance acquisition are required for multiple resistance in the 3-antibiotics model, and treatment strategies using all drugs or restricting some of them may affect the intermediate resistant types differently.

However, an important insight to be gained from the simple model is that infections can be crudely separated to resistant and sensitive to the restricted drug. This decomposition was found useful in analyzing the influence of the model parameters on the emergence of triple resistance.

We found that when bacterial transmission is high, restricting antibiotics is usually not the preferred strategy. Long hospitalization times have a similar effect, and if bacterial transmission is indeed substantial, restricting antibiotics may not be advisable if the patients' mean length of stay is above several days.

Additionally, if antibiotic clearance times are intermediate (less than weeks), or even low but the switch to correct treatment is rapid (less than two days), restricting antibiotics also performs poorly.

We extended our model and considered the interaction between the hospital and the community: where resistance frequencies in the community are affected by the efflux of resistant infections discharged from the hospital, and resistance frequencies among incoming patients are affected by resistance in the community. The weight of the community in the emergence of MDR is hard to determine. The cost of resistance, for instance, could cause a reversion of resistance in bacterial populations that were not recently exposed to a certain antibiotic. As a consequence, the community resistance frequencies would be less influenced by the patient efflux, which is equivalent to low effect of the hospital on the community in our model. However, the significance of the cost of resistance is debatable and perhaps reversion to susceptibility is not often feasible in relevant time scales [39,40].

Therefore, we examined the dynamics with both a relatively high and a negligible influence of the hospital on the community. The community feedback tended to decrease the efficiency of restricting antibiotics. The high usage of only two drugs gave rise to a steady increase in double resistant strains, in the hospital as well as in the community, inevitably treated with the restricted drug and increasing the emergence of triple resistance. Finally, we simulated our model using incoming single and double resistance frequencies estimated from data. Again, restricting antibiotics was usually the less efficient strategy, both with and without the community feedback.

Interestingly, it was recently shown that hospital size affects resistance frequencies [41]. Small hospitals may facilitate extinction of rare resistant strains, and can have substantial effect on the rare infections resistant to the third drug. However, this phenomenon may be mitigated by the resistant strains in the community, and entails further attention.

We focus on mixing a prominent antibiotic prescription strategy, but other treatment strategies may also be considered. Similar results were obtained when running our analyses on the cycling strategy, where drugs are periodically switched every 100 days (S5 Text). Another example is combination therapy, in which patients are treated with several drugs simultaneously. Our model is applicable for this strategy if each of the drugs in the restricted combination have not been abundantly used in the formerly prescribed combinations. To some extent, single drugs in our model (i.e. antibiotics 1, 2, and 3) can be used to describe combinations of drugs, with the addition of a term for partial resistance, due to the overlap of drugs between different combinations. However, the efficiency and toxicity of combination therapy are still a subject of controversy [42]. Moreover, we have previously shown that combination therapy might increase MDR significantly if genetic variation rates are stress-induced [26], suggesting that its use should be carefully considered in such cases.

One could claim that last resort drugs should be reserved for severe infections, to minimize resistance in high risk patients. In contrast, we show that although the usage of the restricted drug can increase its marginal resistance levels, the probability of getting infected by bacteria resistant to all drugs is also decreased. Thus, severe infections with limited treatment options are also minimized.

Our results suggest that the current frequency of resistance to alternative drugs is an important component to be considered when deciding upon the restriction or usage of an antibiotic. Since resistance frequencies can substantially vary between communities [43,44], a sound local estimate of them might be need. This could be obtained by analyzing the records of resistance screens performed on incoming patients, often already available at no additional cost, and perhaps also by sampling commensal bacteria, whose resistance pattern are correlated with the resistance pattern of the infecting bacteria [45]. Analysis of local resistance frequencies could contribute to properly deciding whether to restrict or broadly use certain antibiotics, as well as to monitor and estimate the efficiency of this decision. Moreover, such data has also been proposed for 'informed' strategies: dynamically changing the proportions of drugs prescribed as a function of recent resistance instances within the hospital [46]. Incorporating restricted drug usage with these strategies might lead to even better results at minimizing resistance emergence.

In conclusion, we show that restricted usage of certain drugs, when resistance to the alternative drugs is already present, may sometimes hinder the fight against MDR bacteria. We would like to emphasize that we do not encourage superfluous or increased usage of antibiotics, but rather suggest that changing the proportions of currently used drugs may assist the fight against antibiotic resistance. We hope that our work will promote clinical studies and empirical assessment of the benefits and pitfalls of such a change in treatment policies.

## Supporting Information

S1 Text Approximating equilibrium. (DOCX)

S2 Text Parametric sensitivity analysis. (DOCX)

S3 Text A two antibiotics model. (DOCX)

S4 Text Pairwise comparison of parameters. (DOCX)

S5 Text Results for the cycling strategy. (DOCX)

S1 Table Resistance frequencies of antibiotic pairs. (DOCX)
