# Supplementary material for: Antibiotic Restriction Might Facilitate the Emergence of Multi-drug Resistance
Source: PLoS Comput Biol. 2015 Jun 25;11(6):e1004340. doi: 10.1371/journal.pcbi.1004340 (PMC4481510; doi:10.1371/journal.pcbi.1004340)
Supplement: S1 Text — (DOCX) [file pcbi.1004340.s001.docx]

**S1 Text**

Given the set of odes presented in the main text:

(1A) $X=1-S-R_{1}-R_{2}-R_{3}-R_{1,2}-R_{1,3}-R_{2,3}$

(1B) $\dot{S}=\lambda_{S}-cS+\beta SX-\left( \tau+\gamma\right)S$

(1C) $\dot{R_{i}}=\lambda_{R_{i}}-R_{i}(c+\gamma)+\beta R_{i}X-\tau\left( \chi_{j}+\chi_{k}+\chi_{i}d \right)R_{i}+\tau\chi_{i}Sp_{i}$

(1D) $\dot{R_{j,k}}=\lambda_{R_{j,k}}-R_{j,k}\left( c+\gamma\right)+\beta R_{j,k}X-\tau\left( \chi_{i}+\left( \chi_{j}+\chi_{k} \right)d \right)R_{j,k}+(\chi_{k}+\chi_{j}d\chi_{i,k}^{k})\tau R_{j}p_{k}+{(\chi_{j}+\chi}_{k}d\chi_{i,j}^{j})\tau R_{k}p_{j}$

We will transform the equations 1B-1D, $\dot{V}=f$ to $\frac{\dot{V}}{V+1}=\frac{f}{V+1}$

$$\dot{\frac{S}{S+1}}=\frac{\lambda_{S}}{S+1}+\beta X\frac{S}{1+S}-\left( c+\tau+\gamma\right)\frac{S}{1+S}=\frac{\lambda_{S}}{S+1}+\beta X\left( 1-\frac{1}{1+S} \right)-\left( c+\tau+\gamma\right)\left( 1-\frac{1}{1+S} \right)$$

$$\frac{\dot{R_{i}}}{R_{i}+1}=\frac{\lambda_{R_{i}}}{R_{i}+1}-\left( c+\gamma\right)\frac{R_{i}}{1+R_{i}}+\beta X\frac{R_{i}}{1+R_{i}}-\tau\left( \chi_{j}+\chi_{k}+\chi_{i}d \right)\frac{R_{i}}{1+R_{i}}+\frac{\tau\chi_{i}}{R_{i}+1}Sp_{i}=\frac{\lambda_{R_{i}}}{R_{i}+1}-\left( c+\gamma\right)\left( 1-\frac{R_{i}}{1+R_{i}} \right)+\beta X\left( 1-\frac{R_{i}}{1+R_{i}} \right)-\tau\left( \chi_{j}+\chi_{k}+\chi_{i}d \right)\left( 1-\frac{R_{i}}{1+R_{i}} \right)+\frac{\tau\chi_{i}}{R_{i}+1}Sp_{i}$$

$$\frac{\dot{R_{j,k}}}{R_{j,k}+1}=\frac{\lambda_{R_{j,k}}}{R_{j,k}+1}-\left( c+\gamma\right)\frac{R_{j,k}}{1+R_{j,k}}+\beta X\frac{R_{j,k}}{1+R_{j,k}}-\tau\left( \chi_{i}+\left( \chi_{j}+\chi_{k} \right)d \right)\frac{R_{j,k}}{1+R_{j,k}}+\frac{\chi_{k}\tau}{R_{j,k}+1}R_{j}p_{k}+\frac{\chi_{j}\tau}{R_{j,k}+1}R_{k}p_{j}=\frac{\lambda_{R_{j,k}}}{R_{j,k}+1}-\left( c+\gamma\right)\left( 1-\frac{1}{1+R_{j,k}} \right)+\beta X\left( 1-\frac{1}{1+R_{j,k}} \right)-\tau\left( \chi_{i}+\left( \chi_{j}+\chi_{k} \right)d \right)\left( 1-\frac{1}{1+R_{j,k}} \right)+\frac{\chi_{k}+\chi_{j}d\chi_{i,k}^{k}}{R_{j,k}+1}{\tau R}_{j}p_{k}+\frac{{\chi_{j}+\chi}_{k}d\chi_{i,j}^{j}}{R_{j,k}+1}{\tau R}_{k}p_{j}$$

It was recently shown that the transformation $e^{v(t)}=V\left( t \right)$ can be useful for a first order approximation of the equilibrium of similar dynamic systems, after some algebraic manipulation [1]. However, the approximation holds for $V\left( t \right)\approx1$, so for our set of equations we will define $e^{v(t)}=V\left( t \right)+1$. Now we can use the identity $v\left( t \right)=\ln\left( V\left( t \right)+1 \right)\Rightarrow\frac{\dot{V}}{V+1}=\dot{v}$ and rewrite the equations with our new, lower case variables, and then use the approximation $e^{z}\approx1+z$ :

$$X\approx1-s-r_{1}-r_{2}-r_{3}-r_{1,2}-r_{1,3}-r_{2,3}$$

$$\dot{s} {\approx\lambda}_{S}\left( 1-s \right)-\left( c+\tau+\gamma\right)\left( s \right)+\beta s=s\left( \beta-\lambda_{s}-c-\tau-\gamma\right)+\lambda_{s}$$

$$\dot{r_{i}}\approx r_{i}\left( \beta-\lambda_{R_{i}}-c-\gamma-\tau\left( \chi_{j}+\chi_{k}+\chi_{i}d \right) \right)+\lambda_{R_{i}}+\tau\chi_{i}p_{i}s$$

$$\dot{r_{j,k}}\approx r_{j,k}(\beta-\lambda_{R_{j,k}}-c-\gamma-\tau\left( \chi_{i}+\left( \chi_{j}+\chi_{k} \right)d \right)+\lambda_{R_{j,k}}+\tau({\chi_{j}+\chi}_{k}d\chi_{i,j}^{j})p_{j}r_{k}+\tau(\chi_{k}+\chi_{j}d\chi_{i,k}^{k})p_{k}r_{j}$$

We used the assumption that most patients are uninfected with the specific bacteria we model $(X>\frac{1}{2})$, to achieve a more accurate approximation, by replacing $e^{x}\approx1+x$ with $e^{x}\approx8-\Sigma_{v_{i}\neq x}\left( 1+v_{i} \right)$ (corollary (1)).Note that without loss of generality, this assumption holds for any variable $V$ satisfying $V>\frac{1}{2}$ (of course, there could be only one such variable) and the equations could be easily rewritten for such a case.

The solution for the stable state is simple for the susceptible population:

$$s^{*}=\frac{\lambda_{s}}{-\beta+\lambda_{s}+c+\tau+\gamma}$$

Each of the single resistant strains has a relatively simple solution as well:

$$r_{i}^{*}=\frac{\lambda_{R_{i}}+\tau\chi_{i}p_{i}s^{*}}{\left( -\beta+\lambda_{R_{i}}+c+\gamma+\tau\left( \chi_{j}+\chi_{k}+\chi_{i}d \right) \right)}=\frac{\lambda_{R_{i}}+\frac{\tau\chi_{i}p_{i}\lambda_{s}}{-\beta+\lambda_{s}+c+\tau+\gamma}}{\left( -\beta+\lambda_{R_{i}}+c+\gamma+\tau\left( \chi_{j}+\chi_{k}+\chi_{i}d \right) \right)}$$

The solution for both double resistant strains is more complex:

$$r_{j,k}^{*}=\frac{\lambda_{R_{j,k}}+\tau({\chi_{j}+\chi}_{k}d\chi_{i,j}^{j})p_{j}r_{k}^{*}+\tau(\chi_{k}+\chi_{j}+d\chi_{i,k}^{k})p_{k}r_{j}^{*}}{(-\beta+\lambda_{R_{j,k}}+c+\gamma+\tau\left( \chi_{i}+\left( \chi_{j}+\chi_{k} \right)d \right)}=\frac{\lambda_{R_{j,k}}+\tau\chi_{j}p_{j}\left( \frac{\lambda_{R_{k}}+\frac{\tau(\chi_{k}+\chi_{j}+d\chi_{i,k}^{k})p_{k}\lambda_{s}}{-\beta+\lambda_{s}+c+\tau+\gamma}}{\left( -\beta+\lambda_{R_{k}}+c+\gamma+\tau\left( \chi_{j}+\chi_{i}+\chi_{k}d \right) \right)} \right)+\tau\chi_{k}p_{k}\left( \frac{\lambda_{R_{j}}+\frac{\tau({\chi_{j}+\chi}_{k}+d\chi_{i,j}^{j})p_{j}\lambda_{s}}{-\beta+\lambda_{s}+c+\tau+\gamma}}{\left( -\beta+\lambda_{R_{j}}+c+\gamma+\tau\left( \chi_{k}+\chi_{i}+\chi_{j}d \right) \right)} \right)}{(-\beta+\lambda_{R_{j,k}}+c+\gamma+\tau\left( \chi_{i}+\left( \chi_{j}+\chi_{k} \right)d \right)}$$

A necessary constraint for this approximation is that

$\beta<\lambda_{V}+c+\gamma+\tau*Treatment\left(V\right), \forall V\neq X$. . Where $Treatment\left(V\right)$ is the fraction of patients of class $V$ treated.

Usually, $\lambda_{R_{j,k}}<\lambda_{V} and Treatment\left(R_{j,k}\right)\geq Treatment\left(V\right),\text{ } \forall V\neq X$ and we get that the condition translates to

(1) $\beta<\lambda_{R_{j,k}}+c+\gamma+\tau\left(\chi_{i}+\left(\chi_{j}+\chi_{k}\right)d\right)$

Proof of corollary (I):

We will prove that $e^{x}=8-\text{Σ}_{v_{i}\neq x}e^{v_{i}}\approx8-\text{Σ}_{v_{i}\neq x}\left(1+v_{i}\right)$ is a more accurate approximation than $e^{x}\approx1+x$.

This is equivalent to minimizing the error term:

$$\left|\text{ }e^{x}-\left(1+x\right)\right|\geq\left|\text{ }e^{x}-\left(8-\text{Σ}_{v_{i}\neq x}e^{v_{i}}\right)\right|=\left|\text{ }\left(8-\text{Σ}_{v_{i}\neq x}\left(1+v_{i}\right)\right)-\left(8-\text{Σ}_{v_{i}\neq x}\left(1+v_{i}\right)\right)\right|$$

$$\Leftrightarrow\left|\text{ }e^{x}-\left(1+x\right)\right|\geq\left|\text{Σ}_{v_{i}\neq x}e^{v_{i}}-\text{ Σ}_{v_{i}\neq x}\left(1+v_{i}\right)\right|$$

$$\mathop{\mathop{\Leftrightarrow}\limits_{*} }\limits e^{x}-\left(1+x\right)\geq\text{Σ}_{v_{i}\neq x}e^{v_{i}}-\text{ Σ}_{v_{i}\neq x}\left(1+v_{i}\right)$$

$$\Leftrightarrow e^{x}-\text{Σ}_{v_{i}\neq x}e^{v_{i}}\geq\left(1+x\right)-\text{ Σ}_{v_{i}\neq x}\left(1+v_{i}\right)$$

$$\Leftrightarrow X+1-\text{Σ}_{V_{i}\neq X}V_{i}-7\geq1+\ln\left(1+X\right)-7-\text{Σ}_{V_{i}\neq X}\text{ln}\left(1+V_{i}\right)$$

$$\Leftrightarrow X-\text{Σ}_{V_{i}\neq X}V_{i}\geq\ln\left(1+X\right)-\text{Σ}_{V_{i}\neq X}\text{ln}\left(1+V_{i}\right)$$

However, $\ln\left(1+X\right)-\text{Σ}_{V_{i}\neq X}\ln\left(1+V_{i}\right)\mathop{\leq_{**} }\limits\ln\left(1+X\right)-\ln\left(1+\text{Σ}_{V_{i}\neq X}V_{i}\right)$

In addition $X-\ln\left(1+X\right)\mathop{\geq_{***} }\limits\text{Σ}_{V_{i}\neq X}V_{i}-\ln\left(1+\text{Σ}_{V_{i}\neq X}V_{i}\right)$

$\Rightarrow X-\text{Σ}_{V_{i}\neq X}V_{i}\geq\ln\left(1+X\right)-\ln\left(1+\text{Σ}_{V_{i}\neq X}V_{i}\right)\geq\ln\left(1+X\right)-\Sigma_{V_{i}\neq X}ln\left(1+V_{i}\right)$ *Q.E.D*

$*e^{z}\geq\left(1+z\right) \forall z>0$, $**\log\left(1+\text{z}\right)\text{is concanve},\text{ and therefore subadditive}$, $***\text{Under Assumption I and the fact that} \left(z-\log\left(1+z\right)\right)\text{ is monotonically increasing}.$**References:** 1. Fujii, Kazuyuki. "Comment on" Epidemiological modeling of online social network dynamics"." *arXiv preprint arXiv:1402.1225* (2014).

**
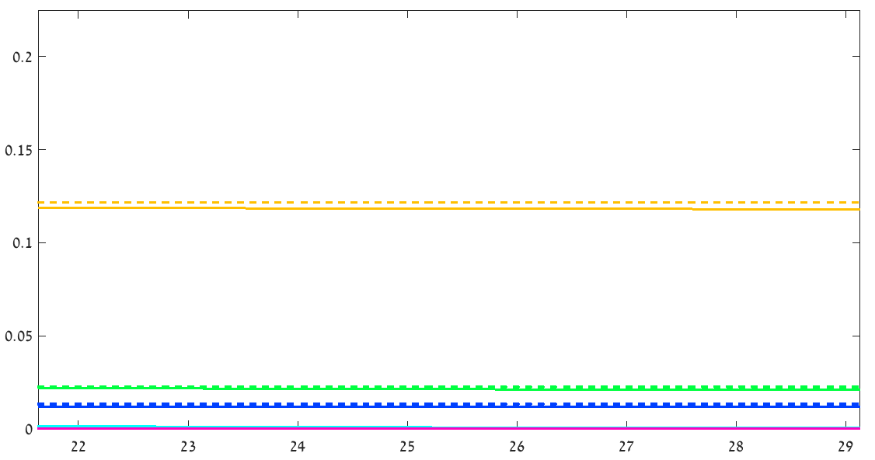

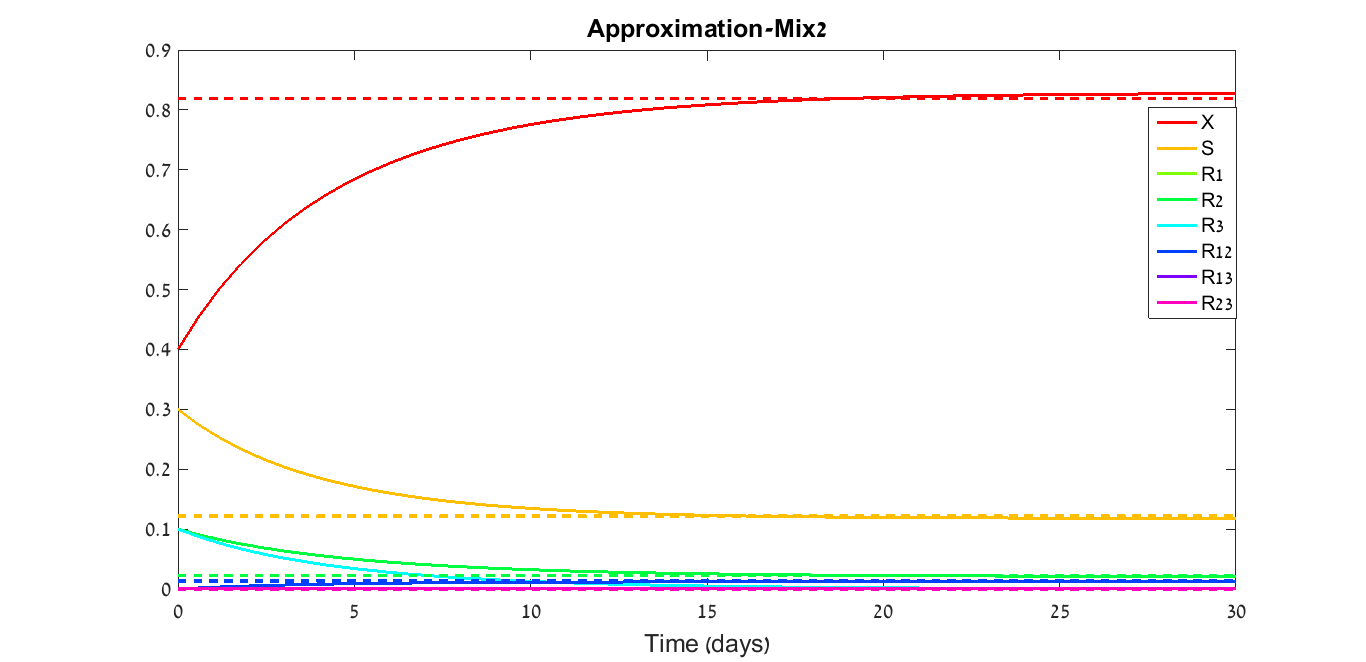
**Below we show the approximation (dashed lines) against numerical integration results (solid curves) for parameters $\begin{matrix} \lambda_{X}=0.07,p_{1}=p_{2}=p_{3}=0.07,c=0.1 ,\beta=0.1,\lambda_{R_{1}}=\lambda_{R_{2}}=0.003,\lambda_{R_{3}}=\lambda_{R_{1,3}}=\lambda_{R_{2,3}}=3*10^{-5}, \\ \lambda_{R_{1,2}}=0.0015,\lambda_{S}=c-\lambda_{X}-\lambda_{R_{1}}-\lambda_{R_{2}}-\lambda_{R_{3}}-\lambda_{R_{1,2}}-\lambda_{R_{1,3}}-\lambda_{R_{2,3}},\gamma=0.03,d=0.778 \end{matrix}$for $mix3$and $mix2$. Embedded plots are enlarged sections of the original plots, made for the reader's convenience.

**
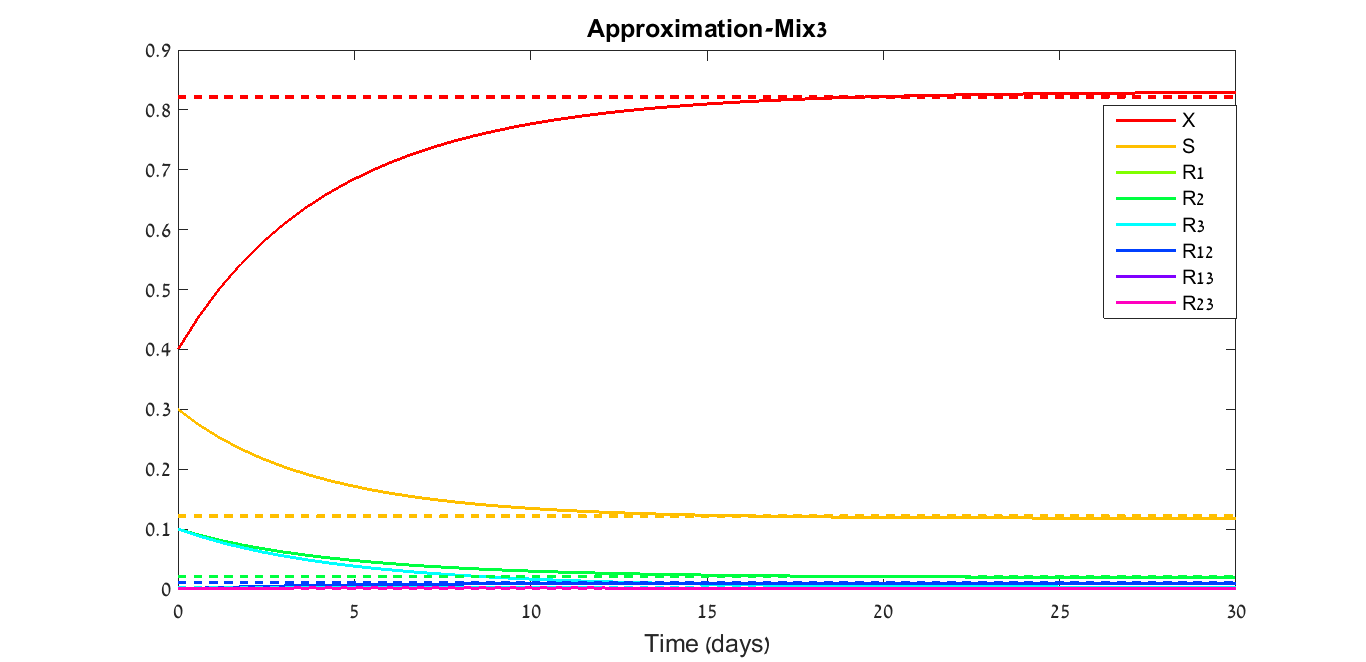

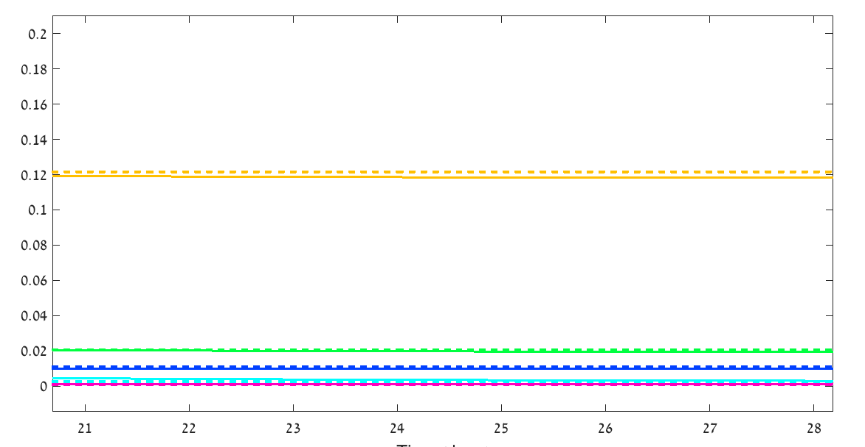
Figure. A**

**Figure. B**
