# Supplementary material for: Antibiotic Restriction Might Facilitate the Emergence of Multi-drug Resistance
Source: PLoS Comput Biol. 2015 Jun 25;11(6):e1004340. doi: 10.1371/journal.pcbi.1004340 (PMC4481510; doi:10.1371/journal.pcbi.1004340)
Supplement: S2 Text — (DOCX) [file pcbi.1004340.s002.docx]

**S2 Text**

We have performed a parametric sensitivity analysis (PSA) for the system, using the direct method. The method is comprised of deriving the model variables relative to the model parameters so that the sensitivity of variable $V$ to a parameter $\alpha$ is defined as $S_{V,\alpha}\left(t\right)=\frac{\partial V\left(t\right)}{\partial\alpha}$. The sensitivity metric chosen to compare various parameters is the time integral$: \bar{S}_{V,\alpha}=\frac{1}{t_{1}-t_{0}}\int_{t_{0}}^{t_{1}} S_{V,\alpha}\left(t\right)dt$.

Sensitivity plots for all strategies are given in Figures S1-S4. Parameters are $f_{R_{1}}= f_{R_{2}}=f_{R_{1,2}}=0.1,\beta=0.3, \lambda_{X}=0.07,p_{1}=p_{2}=p_{3}=0.07,$

$\lambda_{S}=c-\lambda_{X}-\lambda_{R_{1}}-\lambda_{R_{2}}-\lambda_{R_{3}}-\lambda_{R_{1,2}}-\lambda_{R_{1,3}}-\lambda_{R_{2,3}}$. The rest are given in Table 1 in the main text. Similarly to the analytic approximation, even with higher $\beta$ values, the main parameters affecting the resistant strains, and thus our main measures of interest, are the entrance rates.

Parametric sensitivity analysis plots for $mix2$.

Parametric sensitivity analysis plots for $mix3$.

Parametric sensitivity analysis plots for $cyc2$.

Parametric sensitivity analysis plots for $cyc3$.
