# Supplementary material for: Antibiotic Restriction Might Facilitate the Emergence of Multi-drug Resistance
Source: PLoS Comput Biol. 2015 Jun 25;11(6):e1004340. doi: 10.1371/journal.pcbi.1004340 (PMC4481510; doi:10.1371/journal.pcbi.1004340)
Supplement: S3 Text — (DOCX) [file pcbi.1004340.s003.docx]

**S3 Text**

We simulate a reduced version of our model, containing only infections resistant to antibiotics 1 and 2, without the double resistant strain. This is done by setting any variable and parameter values pertaining to antibiotic 3 and to the double resistant infections to zero. The result in the following set of equations:

$$X=1-S-R_{1}-R_{2}$$

We compare the incorrect treatment and the emergence of double resistance for three strategies: using only antibiotic 1 ($\chi_{1}\left(simple\right)=1,\chi_{2}\left(simple\right)=0$), and switching to the second in case of resistance; mixing both antibiotics ($mix2$); and cycling both antibiotics ($cyc2$). The results are plotted below, as a function of the entrance rates of infections resistant to antibiotic 1 (analogously to figures 2 and 4 in the main text, with the same parameter values). Parameters are $f_{R_{1}}= f_{R_{2}}=0.1,\beta=0.3, \lambda_{X}=0.07,p_{1}=p_{2}=p_{3}=0.07,$ $\lambda_{S}=c-\lambda_{X}-\lambda_{R_{1}}-\lambda_{R_{2}}$, and the rest are given at Table 1.
