# Supplementary material for: Antibiotic Restriction Might Facilitate the Emergence of Multi-drug Resistance
Source: PLoS Comput Biol. 2015 Jun 25;11(6):e1004340. doi: 10.1371/journal.pcbi.1004340 (PMC4481510; doi:10.1371/journal.pcbi.1004340)
Supplement: S4 Text — (DOCX) [file pcbi.1004340.s004.docx]

**S4 Text**

**Pairwise comparison of parameters for the mixing strategy.**

We plot the ratio of triple resistance emergence under $mix3$relative to $mix2$ (color scale, left hand panel), for various pairs of parameters. The contribution of $R_{1,2}$ and $R_{1,3}+R_{2,3}$under $mix3$, relative to $R_{1,2}+R_{1,3}+R_{2,3}$ under $mix2$, is presented in the middle and right hand panels, respectively. This enables us to see how parameters affect the common double resistant type ($R_{1,2}$) and the rare double resistance types ($R_{1,3}+R_{2,3}$), and how this is combined to the total triple resistance. The parameters and ranges chosen are:$\text{ 0.01}\leq\beta\leq0.5,\text{ 0.03}\leq\tau\leq0.33,\text{ 0.2}\leq d\leq1,0.033<c<1,\text{ 0.001}\leq p_{1},p_{2},p_{3}\leq0.3$. The rest of the parameters are as in the Figure 2, with $\lambda_{R1,2}=\lambda_{R1}=\lambda_{R2}$. Figure 5 shows the results of Figures 1 and 2 with constant resistance acquisition frequency, i.e. $p_{1}=p_{2}=p_{3}=(1/7*0.07)/\tau$.

**
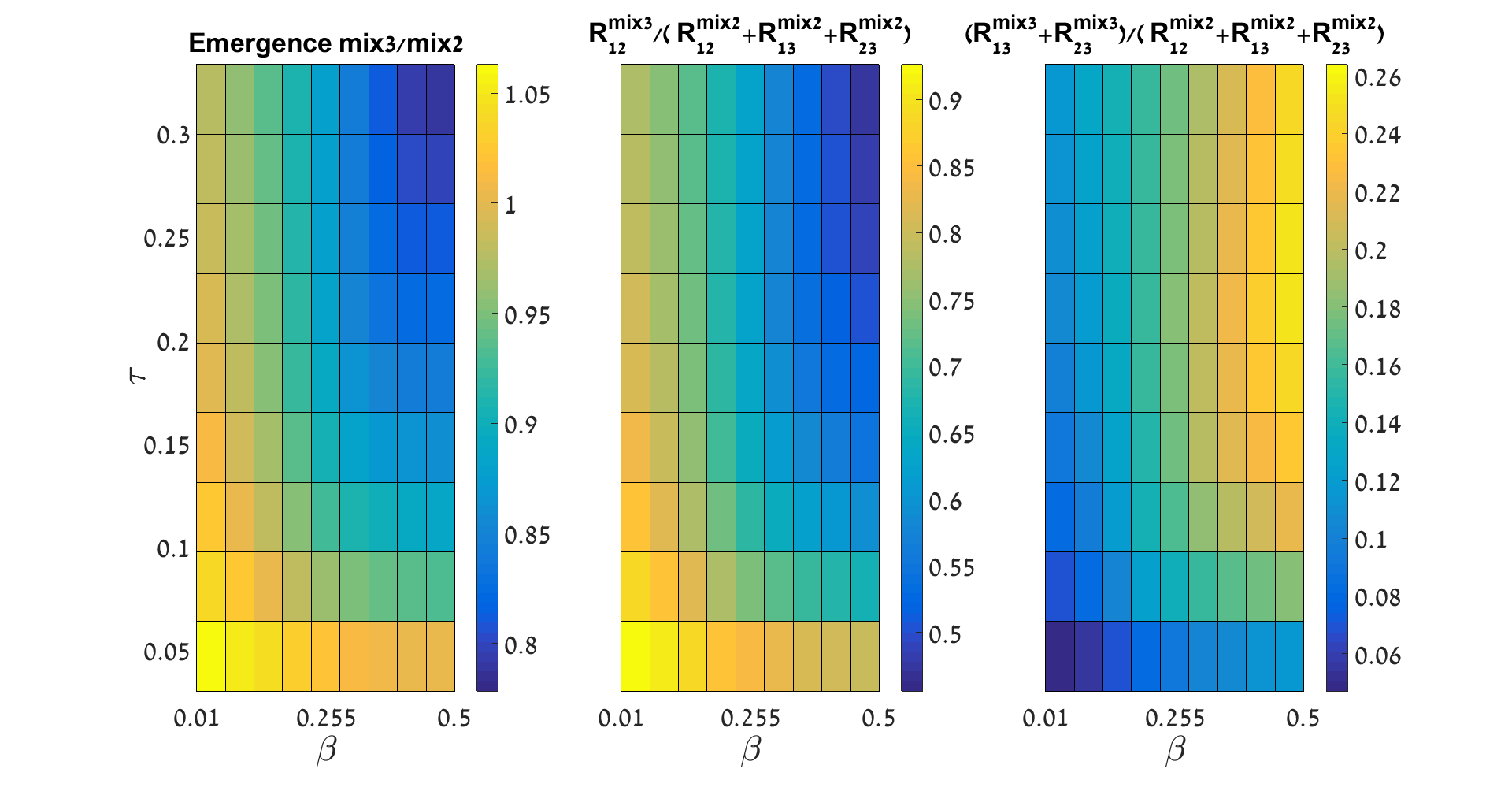
Fig A**

**
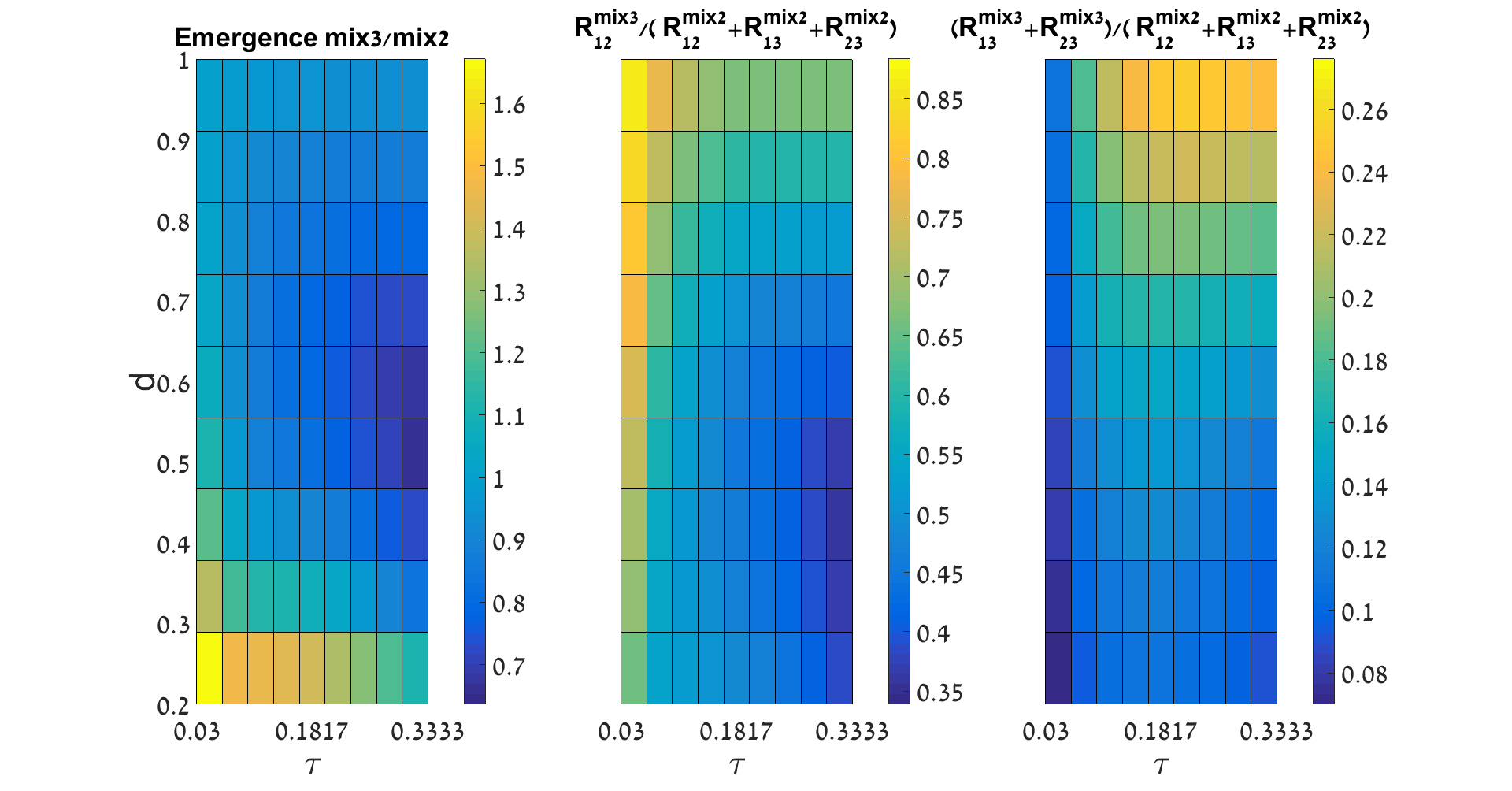
Fig B**

**Fig C**


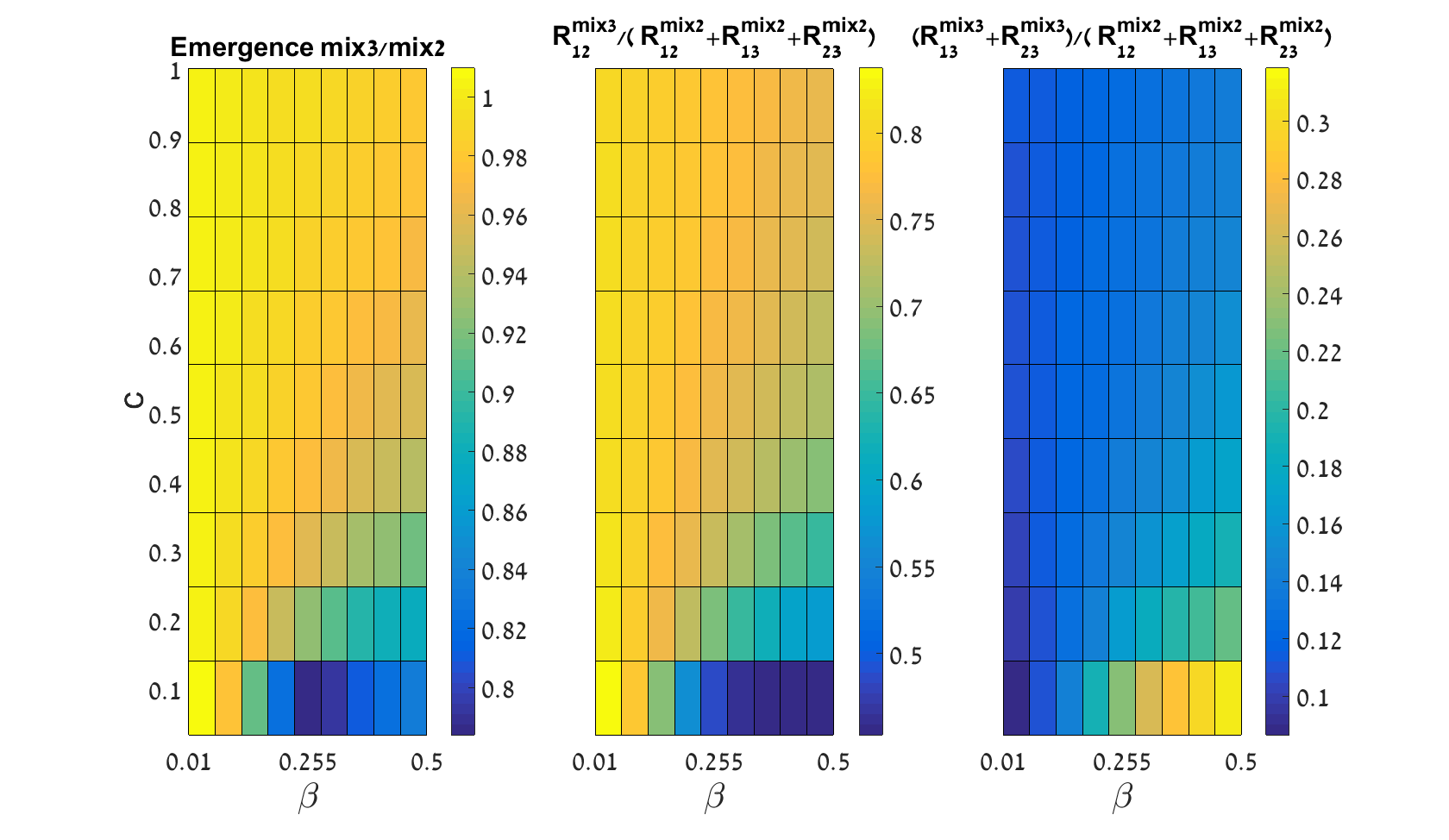

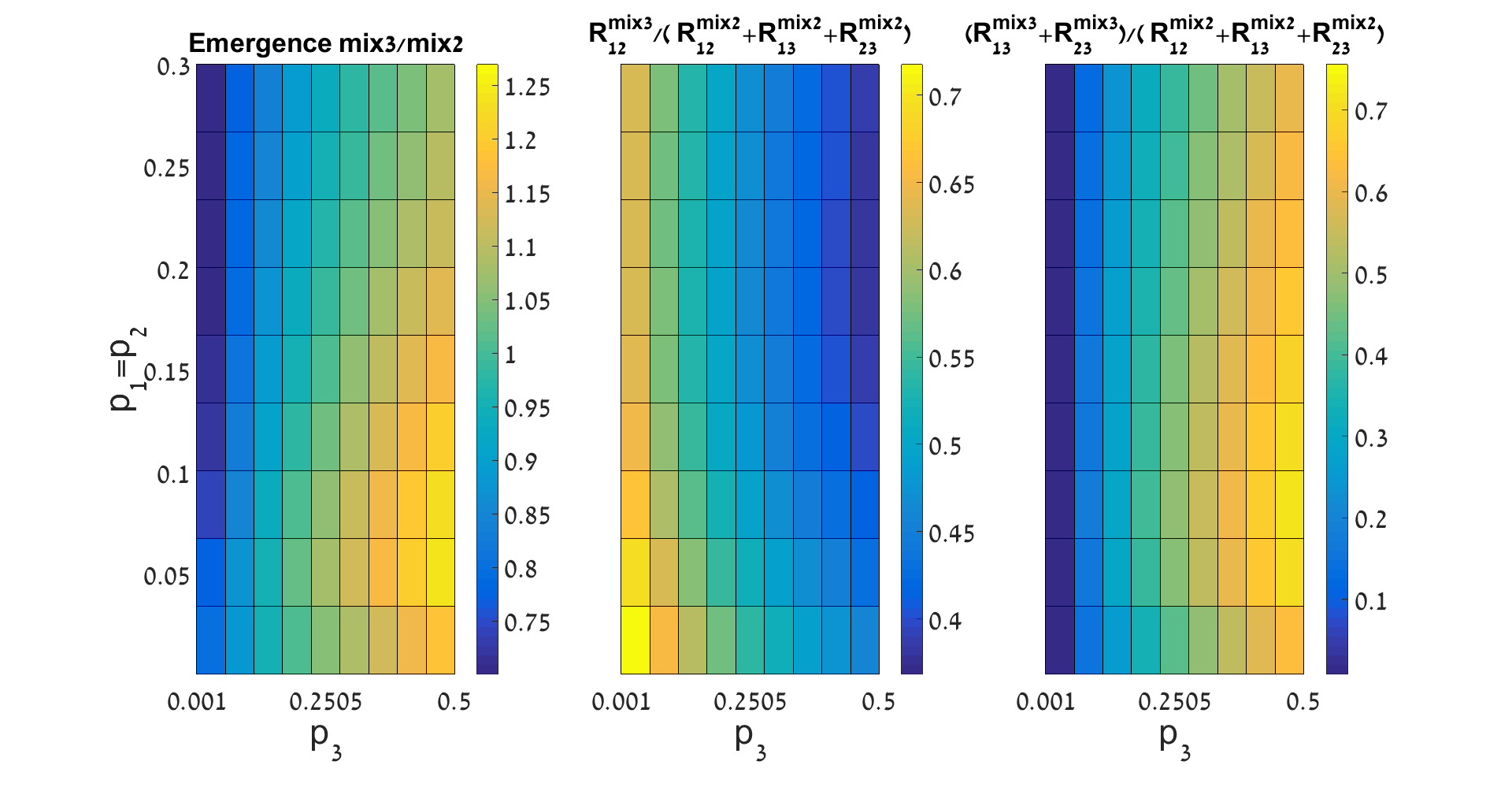
**Fig D**

**Fig E**

**
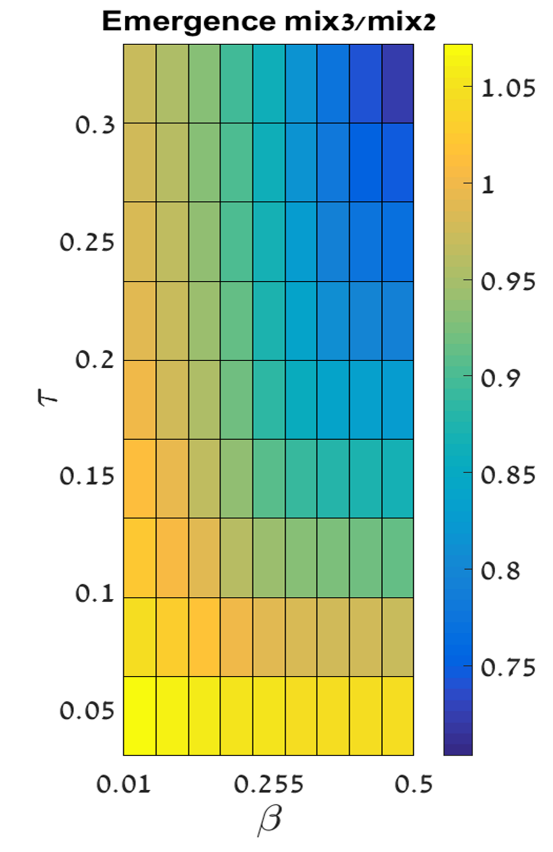

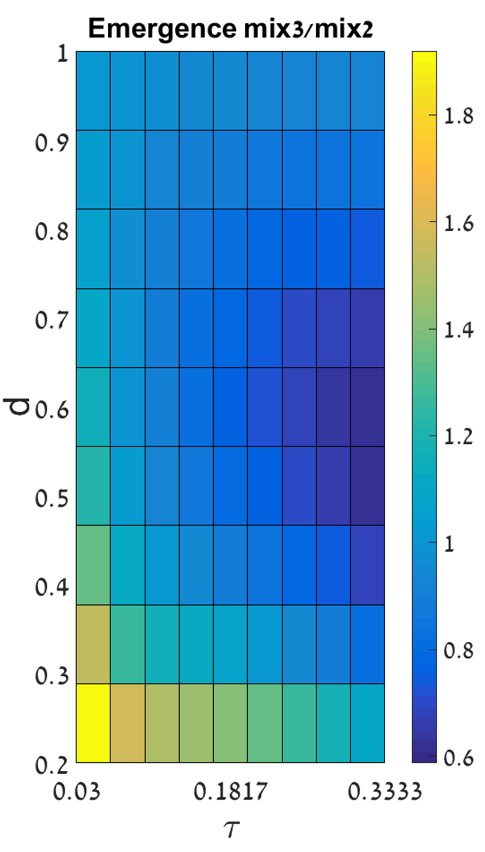
**
