# Supplementary material for: Antibiotic Restriction Might Facilitate the Emergence of Multi-drug Resistance
Source: PLoS Comput Biol. 2015 Jun 25;11(6):e1004340. doi: 10.1371/journal.pcbi.1004340 (PMC4481510; doi:10.1371/journal.pcbi.1004340)

**S5 Text**

This text encompasses the analogous results of our model, obtained for the cycling strategy.

Cycling is the periodic switching between drugs, where at each period of using antibiotic $i$, denoted $\theta_{i}$, only antibiotic $i$will be prescribed. Similarly to mixing, we can cycle two drugs, denoted $cyc2$, and reserve the third: $\chi_{1}\left(t,cyc2\right)=I_{t\in\theta_{1}}, \chi_{2}\left(t,cyc2\right)=1-\chi_{1}\left(t,cyc2\right), \chi_{3}\left(t,cyc2\right)=0$(where $I_{t\in\theta_{i}}=\left\{\begin{matrix} 1\text{ } if t\in\theta_{i} \\ 0 \text{ }else \end{matrix}\right.$);

Cycling all three drugs will be denoted $cyc3$: $\chi_{1}\left(t,cyc3\right)=I_{t\in\theta_{1}},\chi_{2}\left(t,cyc3\right)=I_{t\in\theta_{2}}, \chi_{3}\left(t,cyc3\right)=I_{t\in\theta_{3}}$.

The figures are denoted as Figure XS, for the relevant Figure X in the main text. Parameters are $f_{R_{1}}= f_{R_{2}}=0.1,\beta=0.3, \lambda_{X}=0.07,p_{1}=p_{2}=p_{3}=0.07$ $\lambda_{S}=c-\lambda_{X}-\lambda_{R_{1}}-\lambda_{R_{2}}-\lambda_{R_{3}}-\lambda_{R_{1,2}}-\lambda_{R_{1,3}}-\lambda_{R_{2,3}}$, and the rest are given at Table 1. Cycling is performed for periods of 100 days.

**Figure 2S**


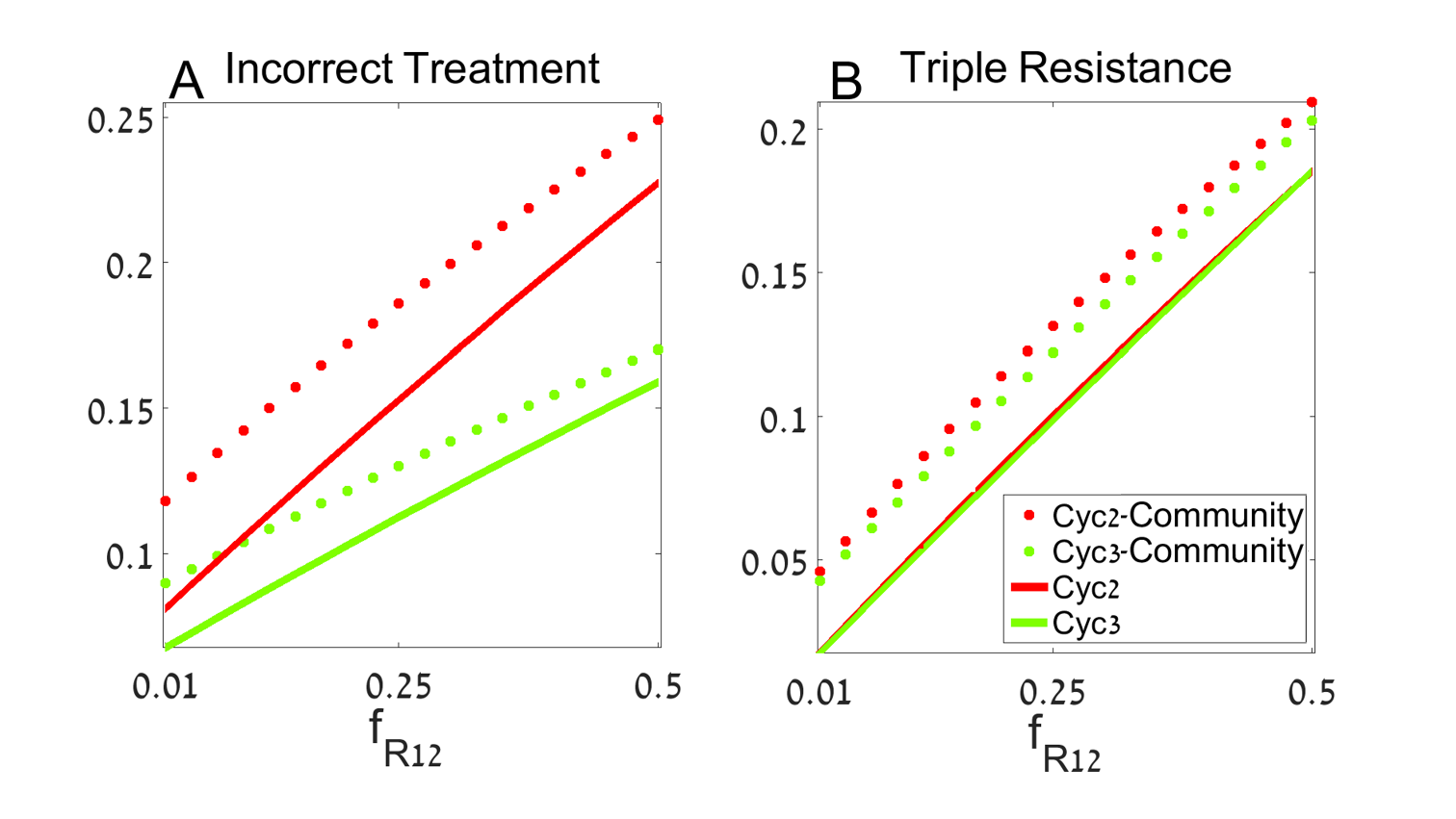


**Figure 3S**


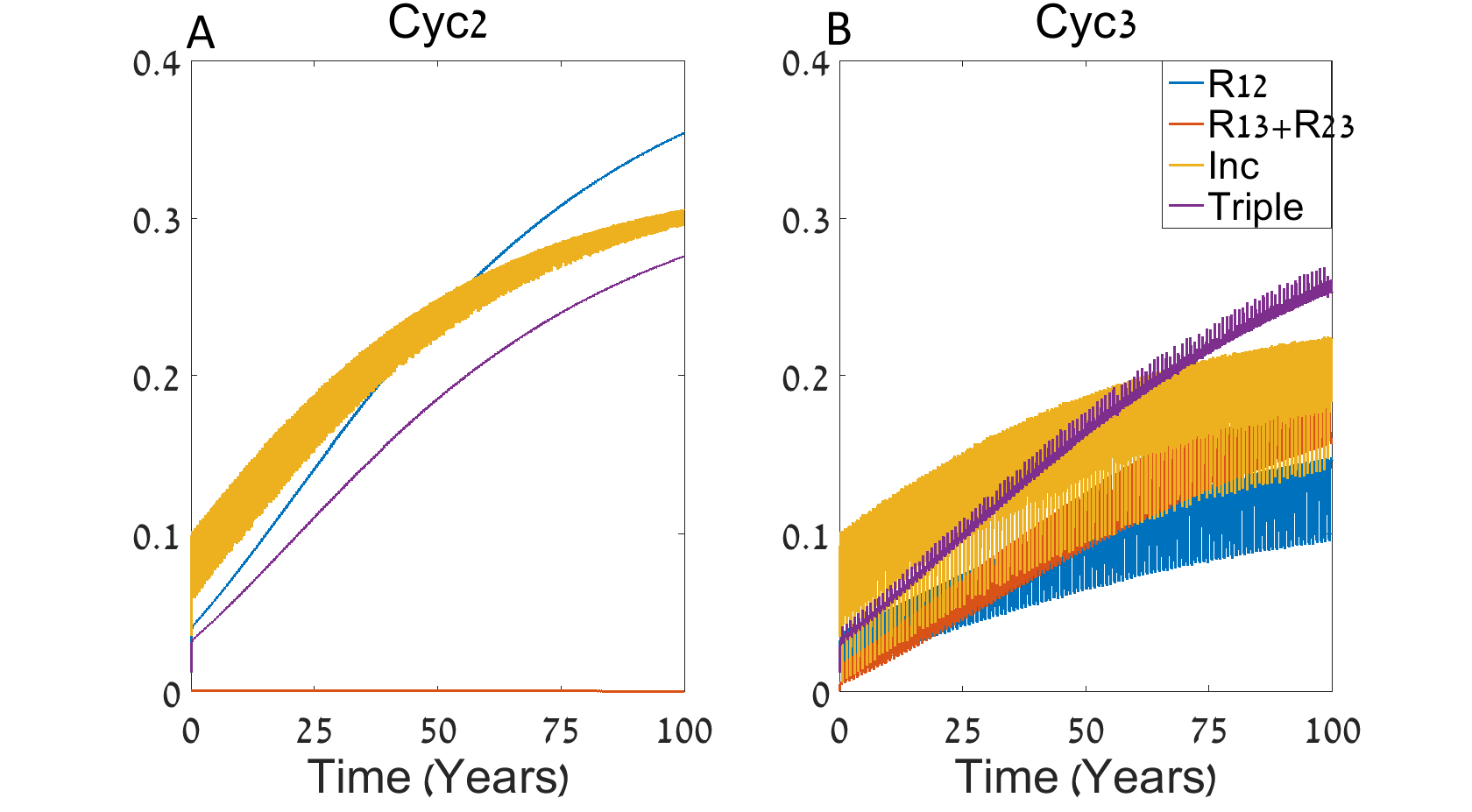


**Figure 4S**


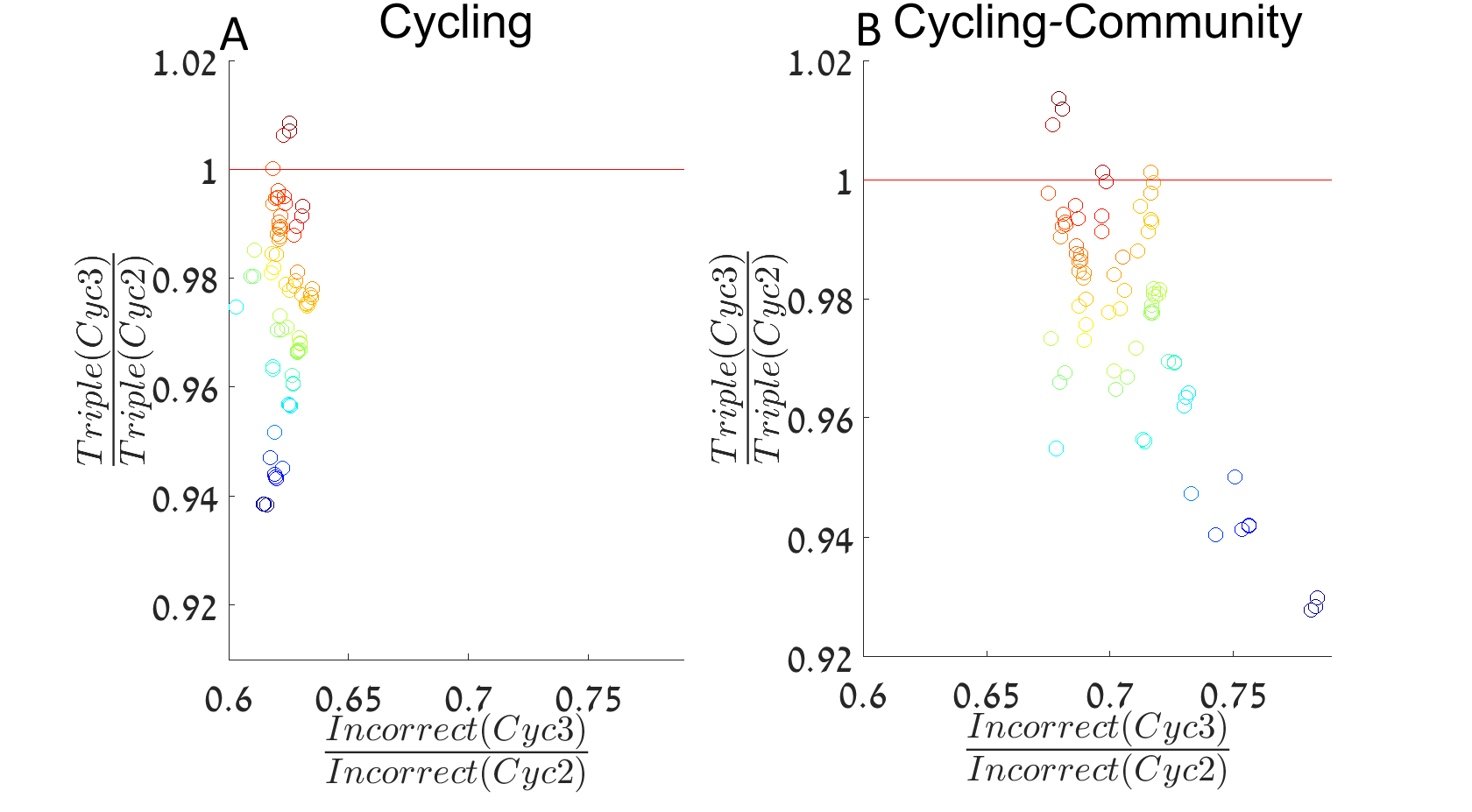

Supplement: S5 Text — (DOCX) [file pcbi.1004340.s005.docx]
