# Supplementary material for: Antibiotic Restriction Might Facilitate the Emergence of Multi-drug Resistance
Source: PLoS Comput Biol. 2015 Jun 25;11(6):e1004340. doi: 10.1371/journal.pcbi.1004340 (PMC4481510; doi:10.1371/journal.pcbi.1004340)
Supplement: S1 Table — (DOCX) [file pcbi.1004340.s006.docx]

**S1 Table:**

Resistance frequencies of antibiotic pairs. N is the number of cultures in which both antibiotics were tested for resistance. $\hat{f}_{R_{1}},\hat{f}_{R_{2}},\hat{f}_{R_{1,2}}$are calculated by the fraction of cultures resistant to antibiotic 1 alone, antibiotic 2 alone, and to both antibiotics, respectively. Abbreviations are: A/S, Ampicillin/sulbactam; A/C, Amoxicillin/Clavulanic Acid; T/C , Ticarcillin/clavulanic acid; T/S, Trimethoprim/sulfamethoxazole; Ceft, Ceftriaxone; Cefo, Cefotaxime; Levo, Levofloxacin; P/t, Piperacillin/tazobactam; Tazo, Tazocin; Mero, Meropenem; Imip, Imipenem; Erta, Ertapenem; Chlo, Chloramphenicol; Cefu, Cefuroxime; Cefe, Cefepime.

| Antibiotic 1 | Antibiotic 2 | N | $\hat{f}_{R_{1}}$ | $\hat{f}_{R_{2}}$ | $\hat{f}_{R_{1,2}}$ |
| --- | --- | --- | --- | --- | --- |
| A/C | A/S | 464 | 0.068966 | 0.090517 | 0.459052 |
| A/C | Cefo | 395 | 0.270886 | 0.053165 | 0.243038 |
| A/C | Ceft | 484 | 0.241736 | 0.070248 | 0.280992 |
| A/C | Cefu | 485 | 0.179381 | 0.098969 | 0.34433 |
| A/C | Erta | 467 | 0.503212 | 0.004283 | 0.023555 |
| A/C | Imip | 472 | 0.504237 | 0.002119 | 0.029661 |
| A/C | Levo | 53 | 0.226415 | 0.09434 | 0.45283 |
| A/C | Mero | 456 | 0.508772 | 0.002193 | 0.024123 |
| A/C | P/T | 75 | 0.453333 | 0.013333 | 0.173333 |
| A/C | T/S | 482 | 0.236515 | 0.126556 | 0.288382 |
| A/C | Tazo | 398 | 0.404523 | 0.005025 | 0.110553 |
| A/C | T/C | 447 | 0.165548 | 0.06264 | 0.357942 |
| A/S | Cefo | 392 | 0.280612 | 0.053571 | 0.242347 |
| A/S | Ceft | 463 | 0.263499 | 0.062635 | 0.285097 |
| A/S | Cefu | 464 | 0.185345 | 0.079741 | 0.364224 |
| A/S | Erta | 450 | 0.548889 | 0.011111 | 0.015556 |
| A/S | Imip | 461 | 0.529284 | 0.010846 | 0.019523 |
| A/S | Levo | 54 | 0.222222 | 0.074074 | 0.481481 |
| A/S | Mero | 452 | 0.530973 | 0.011062 | 0.013274 |
| A/S | P/T | 69 | 0.521739 | 0.028986 | 0.15942 |
| A/S | T/S | 463 | 0.241901 | 0.107991 | 0.308855 |
| A/S | Tazo | 394 | 0.426396 | 0.015228 | 0.098985 |
| A/S | T/C | 442 | 0.183258 | 0.061086 | 0.359729 |
| Cefo | Ceft | 394 | 0.005076 | 0.007614 | 0.28934 |
| Cefo | Cefu | 395 | 0 | 0.098734 | 0.296203 |
| Cefo | Erta | 386 | 0.266839 | 0.002591 | 0.025907 |
| Cefo | Imip | 393 | 0.274809 | 0.002545 | 0.020356 |
| Cefo | Mero | 395 | 0.273418 | 0 | 0.022785 |
| Cefo | T/S | 394 | 0.07868 | 0.185279 | 0.218274 |
| Cefo | Tazo | 394 | 0.21066 | 0.030457 | 0.083756 |
| Cefo | T/C | 387 | 0.080103 | 0.206718 | 0.21447 |
| Ceft | Cefu | 492 | 0.004065 | 0.093496 | 0.341463 |
| Ceft | Erta | 467 | 0.314775 | 0.006424 | 0.023555 |
| Ceft | Imip | 472 | 0.32839 | 0.002119 | 0.029661 |
| Ceft | Levo | 91 | 0.076923 | 0.043956 | 0.285714 |
| Ceft | Mero | 456 | 0.317982 | 0.004386 | 0.024123 |
| Ceft | P/T | 75 | 0.48 | 0.013333 | 0.173333 |
| Ceft | T/S | 489 | 0.09816 | 0.169734 | 0.249489 |
| Ceft | Tazo | 396 | 0.212121 | 0.030303 | 0.085859 |
| Ceft | T/C | 447 | 0.111857 | 0.192394 | 0.228188 |
| Cefu | Erta | 468 | 0.410256 | 0.004274 | 0.025641 |
| Cefu | Imip | 471 | 0.418259 | 0 | 0.031847 |
| Cefu | Levo | 57 | 0.192982 | 0.035088 | 0.491228 |
| Cefu | Mero | 457 | 0.413567 | 0.002188 | 0.026258 |
| Cefu | P/T | 75 | 0.546667 | 0.013333 | 0.173333 |
| Cefu | T/S | 489 | 0.171779 | 0.147239 | 0.267894 |
| Cefu | Tazo | 398 | 0.301508 | 0.020101 | 0.09799 |
| Cefu | T/C | 448 | 0.176339 | 0.158482 | 0.261161 |
| Chlo | T/S | 427 | 0.156909 | 0.149883 | 0.262295 |
| Erta | Imip | 455 | 0.006593 | 0.006593 | 0.021978 |
| Erta | Levo | 50 | 0 | 0.52 | 0.02 |
| Erta | Mero | 442 | 0.004525 | 0 | 0.024887 |
| Erta | P/T | 69 | 0 | 0.144928 | 0.014493 |
| Erta | T/S | 466 | 0.002146 | 0.386266 | 0.027897 |
| Erta | Tazo | 388 | 0.007732 | 0.095361 | 0.020619 |
| Erta | T/C | 433 | 0.004619 | 0.401848 | 0.020785 |
| Imip | Levo | 52 | 0 | 0.461538 | 0.096154 |
| Imip | Mero | 459 | 0.006536 | 0.002179 | 0.023965 |
| Imip | P/T | 77 | 0.025974 | 0.142857 | 0.038961 |
| Imip | T/S | 470 | 0.002128 | 0.387234 | 0.029787 |
| Imip | Tazo | 437 | 0.009153 | 0.093822 | 0.022883 |
| Imip | T/C | 487 | 0.010267 | 0.386037 | 0.026694 |
| Levo | Mero | 55 | 0.490909 | 0 | 0.054545 |
| Levo | P/T | 50 | 0.42 | 0.06 | 0.1 |
| Levo | T/S | 60 | 0.166667 | 0.1 | 0.35 |
| Mero | P/T | 59 | 0 | 0.118644 | 0.033898 |
| Mero | T/S | 457 | 0 | 0.389497 | 0.028446 |
| Mero | Tazo | 400 | 0.0025 | 0.0975 | 0.02 |
| Mero | T/C | 447 | 0.002237 | 0.402685 | 0.022371 |
| P/T | T/S | 75 | 0.053333 | 0.333333 | 0.133333 |
| P/T | T/C | 59 | 0.016949 | 0.305085 | 0.118644 |
| T/S | Tazo | 397 | 0.322418 | 0.037783 | 0.080605 |
| T/S | T/C | 454 | 0.15859 | 0.165198 | 0.262115 |
| Tazo | T/C | 427 | 0.002342 | 0.29274 | 0.112412 |
